# Reliability and Validity of Seven Feline Behavior and Personality Traits

**DOI:** 10.3390/ani11071991

**Published:** 2021-07-02

**Authors:** Salla Mikkola, Milla Salonen, Emma Hakanen, Sini Sulkama, Hannes Lohi

**Affiliations:** 1 Department of Veterinary Biosciences, University of Helsinki, 00014 Helsinki, Finland; salla.mikkola@helsinki.fi (S.M.); milla.ahola@helsinki.fi (M.S.); emma.hakanen@helsinki.fi (E.H.); sini.sulkama@helsinki.fi (S.S.); 2 Department of Medical and Clinical Genetics, University of Helsinki, 00014 Helsinki, Finland; 3 Folkhälsan Research Center, 00014 Helsinki, Finland

**Keywords:** cat personality, *Felis silvestris catus*, temperament, test–retest reliability, inter-rater reliability, convergent validity, discriminant validity, personality

## Abstract

**Simple Summary:**

Cats have personalities, just like humans and other animals, with stable behavior differences between individuals. Identification of a cat’s personality type is important as cats with different personalities have different environmental needs to reach a good life quality. For example, active individuals may need more enrichment, such as playing, than less active individuals, and fearful cats may benefit from extra hiding places and owners’ peaceful lifestyle. Cats are popular pet animals, but their personality has been little studied. In addition, the majority of these studies used behavior questionnaires that have not been validated. Thus, we studied cat personality and behavior by collecting a large dataset of over 4300 cats with an online questionnaire and studied its validity and reliability. Feline personality and behavior included seven traits: fearfulness, activity/playfulness, aggression toward humans, sociability toward humans, sociability toward cats, excessive grooming and litterbox issues. Breeds differed in all traits, and the questionnaire was reliable and valid. Our findings indicate that owner-completed questionnaires are valid sources of behavior data, and that some personality traits are more common in specific cat breeds. Breed differences, however, should be examined with more complex models, taking other factors, such as the age of the cat, into account.

**Abstract:**

Domestic cats are popular pets, and they have personalities, with stable behavior differences between individuals. Lately, feline behavior and personality have been studied with different approaches, for example, with owner-completed questionnaires. The majority of these studies, however, lack a sufficient validation and reliability assessment of the questionnaires used. We designed an online feline behavior and personality questionnaire to collect cat behavior data from their owners. Then, we ran a factor analysis to study the structure of personality and behavior in a dataset of over 4300 cats. For validation, we studied the internal consistency, test–retest reliability, inter-rater reliability, convergent validity and discriminant validity of this questionnaire and extracted factors. In addition, we briefly examined breed differences in the seven discovered factors: fearfulness, activity/playfulness, aggression toward humans, sociability toward humans, sociability toward cats, excessive grooming and litterbox issues. Most of the rank ordering of breeds within each trait paralleled what has been found in previous studies. The validity and reliability of the questionnaire and factors were good, strengthening owner-completed questionnaires as a method to collect behavioral data from pet animals. Breed differences suggest a genetic background for personality. However, these differences should be studied further with multidimensional models, including environmental and biological variables.

## 1. Introduction

Personality can be defined as interindividual differences in behavior which are stable over time and in distinct contexts [1]. Personality is influenced by genetics, the environment and their interactions [2]. Personality differences between individuals have been found in several species, including domestic cats [3]. In cats, stable individual differences emerge before weaning [4]. Importantly, knowing the personality type of a cat can be used to enhance its welfare [5,6]: for example, fearful cats may benefit from additional hiding places [2]. In addition, owners are more satisfied with their pets if their pet’s personality matches their own [7]. As a result, matching the personalities of people and cats has been utilized, for example, in shelter adoption processes [8]. Furthermore, a cat’s personality may also affect its suitability for families with children [9].

Feline personality has been studied with various methods. In some studies, veterinarians ranked cat breeds based on their professional experiences of seeing, handling and treating cats of different breeds in the clinic [10,11]. Other researchers have used ratings or behavioral observations of cats’ caretakers in shelters or in laboratory colonies [12,13] or behavioral tests [2]. Finally, many studies have utilized owner-completed questionnaires. In these studies, owners are asked to rate their cat’s behavior in different situations or with a set of adjectives.

Previous feline personality studies have found one to six main personality dimensions [2], which often somewhat differ between studies. In an extensive review about feline personality, Travnik and colleagues [2] named some of the dimensions as friendliness, aggressiveness, boldness, openness, activity, impulsiveness and dominance. Previous studies have labeled these traits variably. The Meet Your Match^®^ Feline-ality™ protocol represents a cat’s personality with two main dimensions comparable to the shy–bold axis and friendliness toward humans [8,14]. “The Feline Five” [15] resembles the human Big Five personality structure [16], with the five dimensions labeled as agreeableness, dominance, extraversion, impulsiveness and neuroticism [15]. Ha and Ha [17] found five factors as well, cat social, active, human nonsocial, human aggressive and intense, although they also suggested the existence of a sixth factor. Bennett and colleagues [18] found six dimensions, which they named playfulness, nervousness, amiability, dominance, demandingness and gullibility. Duffy and colleagues [19] found 23 factors for feline personality and problematic behavior.

These previous studies utilized questionnaires, which are a practical and fast method to collect behavior data [4]. Questionnaire answers agree with behavioral observations [20], but still, they are more subjective than direct observations [2], making the validation of questionnaires undeniably important [21]. Internal consistency is the most studied measure in feline survey studies (e.g., [15,18,19,22,23]). Inter-rater reliability was inspected in the studies of Gartner and colleagues [12], Feaver and colleagues [13] and Turner and colleagues [24], and test–retest reliability was assessed in the study of Arahori and colleagues [23]. In addition to internal consistency, Duffy and colleagues [19] also inspected convergent and discriminant validity. Several feline personality studies, however, lack validation of any kind, and some have been validated just partly [21].

In this study, we examined feline personality and behavior by utilizing the accumulated knowledge of cat owners. For this, we used an online questionnaire where owners rated their cats’ responses in several situations using a Likert scale, which is widely used in feline personality studies [2]. The structure of our questionnaire was similar to Duffy and colleagues’ [19] and, thus, included problematic behavior as well. Furthermore, we evaluated our questionnaire’s reliability in time and between observers, convergent and discriminant validity and internal consistency. In addition, we briefly examined breed differences in personality and behavior.

## 2. Methods

### 2.1. Questionnaire

We developed the feline behavior and personality questionnaire based on previous behavior questionnaires, mostly Fe-BARQ (Feline Behavioral Assessment and Research Questionnaire) [19]. We modified the questionnaire content to be more suitable for the Finnish cat population (for example, in Finland, declawing and selling cats in pet shops are illegal). The questionnaire included three sections: behavior, background and health (Supplementary File: Feline personality and behavior questionnaire). We invited nine cat behavior, welfare and health experts to review and comment on the questionnaire to improve its quality. This panel included people with diverse backgrounds, for example, veterinarians, behavior consultants, trainers, members of the animal welfare committee and breeders. After modifying the questionnaire based on the panel discussion, the behavior section included 138 statements in a randomized order. Answering options for these statements were “strongly disagree”, “somewhat disagree”, “neither agree nor disagree”, “somewhat agree”, “strongly agree” and “I do not know”. In addition, we asked whether the owner feels that their cat has problematic behavior, with answering options “no”, “a little”, “some” and “a lot”.

We published the questionnaire in March 2019 on the Petsofi platform [25]. Pet owners provided basic information about themselves and their pets when registering to Petsofi. The mandatory information included owners’ email address, first name, last name and country. In addition, they could fill in their address, official name and nickname of their pet, their pet’s gender, breed, coat color, date of birth, country of birth, breeder, registry ID, registry (FIFE, CFA, TICA or WCF) and chip ID and the main activity of their pet.

### 2.2. Subjects

**Figure 1:**
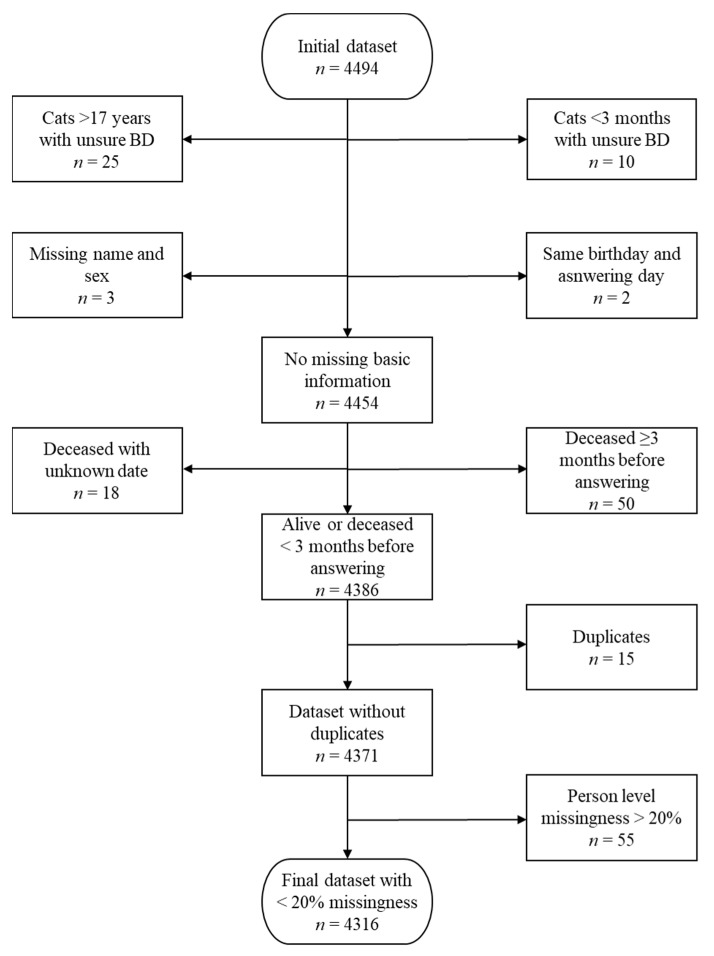
Flow chart of the study population and sample size in feline behavior and personality study. BD = birthdate.

We prepared and filtered the data before analyses. First, we excluded very old (over 17 years) and very young (under three months) cats whose ages were impossible to verify (Figure 1). We also excluded two cats whose birthday was the same day as the answering day. Second, we excluded cats whose owners did not report either the cat’s sex or name. Third, we excluded cats that were deceased for over three months before answering, and cats who had been marked as deceased, but the death date was not reported. Fourth, we removed duplicate cats. We left the more complete answer, or if both were equally completed, we used the more recent answer. Not all owners reported their cat’s sex, and we tried to contact these owners via email. Owners of three cats did not answer, and thus we classified these cats as male/female based on their nicknames. Last, we removed individuals that had more than 20% of missing values in the behavior section, leading to the final sample size of 4316 cats.

The study population included cats from 56 different breeds, house cats and mixed breed cats, which were grouped into 26 groups. The house cat group included house cats (non-purebred cats with a purebred ancestor or apparent breed-specific characteristics, for example, colorpoint color) and mixed breed cats. We handled cats without breed information (n = 280) as house cats. For breed groups American Curl, Bengal, British, Oriental, Persian and Exotic and Siamese and Balinese, short- and long-haired variants were combined. The Oriental group also included Snowshoe, the Siamese and Balinese group Seychellois and the Persian and Exotic group Himalayan, as these breeds are closely related [26,27,28]. In addition, we grouped European and American Burmese into a group named Burmese, as they are genetically closely related breeds, and some cat registries, such as The International Cat Association (TICA), do not even recognize them as separate breeds [29]. We also united Siberian and Neva Masquerade into a group named Siberian and Neva Masquerade. Neva Masquerade is basically a pointed colored Siberian, and, for example, TICA does not separate these two breeds [30]. Furthermore, Sphynx and Devon Rex were grouped, as these breeds are genetically closely related [28]. We also examined landrace cats, which are cats derived from locally adapted Finnish cat populations and do not have breed ancestry. Although short- and long-haired landrace cats are born in the same litters, we wanted to study them separately as their sample sizes enabled that. The other breed group included breeds which were not easily grouped with other breed groups and had less than 21 individuals.

During the last months of data collection, we contacted owners who had participated in the study 1–3 months ago via email. We asked them to answer the questionnaire’s behavior and personality section again (for test–retest reliability) or ask an adult person living in the same household to answer the behavior and personality section about the same cat (for inter-rater reliability). In addition to behavior and personality questions, we asked how long the other respondent had known the cat. The answering options were “less than 3 months”, “3–6 months”, “6 months to 1 year”, “1–5 years” and “over 5 years”. The second respondents also had to declare that they did not discuss the cat’s behavior with the first owner when filling in the questionnaire.

### 2.3. Convergent and Discriminant Validity

We formed 25 hypotheses based on the previous literature to evaluate the convergent validity of extracted factors (Supplementary Table S1). We hypothesized, for example, that female cats are more fearful [10,31] and have less litterbox issues than males [10,32,33]. We also hypothesized that older cats are less active [18,19] and less cat social [31,34] than younger cats. In addition, we hypothesized that cats living with conspecifics are less aggressive toward humans [31,34,35], but have more litterbox issues [36]. We also expected to see more excessive grooming in Burmese and Oriental breeds than Siberians, Neva Masquerades and Norwegian Forest cats [37]. Furthermore, we expected cats whose owners report problematic behavior to have higher scores in fearfulness, aggression toward humans, excessive grooming and litterbox issues than cats without problematic behavior [19]. For this, we compared cats without reported problematic behavior to cats with some or a lot of problematic behavior. For discriminant validity, we examined correlations between factors. We expected that factors which should not be correlated do not correlate.

### 2.4. Statistical Analyses

#### 2.4.1. Factor Analysis

We conducted an explanatory factor analysis to reduce the high number of questions into a smaller number of biologically meaningful traits. Firstly, we excluded questions with more than 24.1% of missing responses (n = 20). After this, we excluded individuals with more than 20% of missing responses (n = 55), leading to the final sample size of 4316 cats (Figure 1). Then, we tested the appropriateness of our dataset for explanatory factor analysis with the Kaiser–Meyer–Olkin test for sampling adequacy (KMO = 0.90) and with Bartlett’s test of sphericity (*p* < 0.0001) from the package psych [38]. The same package was used in later analyses as well. All analyses were conducted using R version 4.0.3 [39].

We used polychoric correlation matrices, as the answers were on a Likert scale, and mean imputation. We decided not to use rotation, as it makes further use of the factor scores more difficult. To determine the optimal number of factors, we used the scree test, parallel analysis and Velicer’s minimum average partial (MAP) test. In addition, we extracted all possible structures (Goldberg’s hierarchical tree) from 1 to 11 factors to evaluate the factor structure. We also compared the root mean square error of approximation (RMSEA) and the Tucker–Lewis index between possible structures.

#### 2.4.2. Internal Consistency, Test–Retest Reliability and Inter-Rater Reliability

We evaluated the factors’ internal consistency with Cronbach’s alpha and Guttman’s lambda 6 using the package psych [38]. To estimate test–retest reliability, we studied correlations between the first and second times of answering, both for individual items and factors. Similarly, we calculated the inter-rater reliabilities both for items and factors with intraclass correlation coefficients.

#### 2.4.3. Convergent and Discriminant Validity

Before examining the validity of the questionnaire, we removed unreliable items based on their test–retest and inter-rater reliabilities. Then, we removed non-loading items (all loadings < 0.3) one by one, constantly re-running the factor analysis after removing an item [40]. After this, we estimated the internal consistency, test–retest reliability and inter-rater reliability for the final factor structure and calculated scores for all individual cats using the “tenBerge” estimation method.

We utilized the factor scores in convergent validity testing. We used Pearson correlations for continuous variables and point-biserial correlations for class variables to calculate the validity coefficients. All *p*-values were corrected for the false discovery rate (FDR).

#### 2.4.4. Breed Differences

We conducted Kruskal–Wallis tests to examine whether breeds differ in factor scores. The significance cut-off *p*-value was set at *p* < 0.05, and *p*-values were corrected for the false discovery rate (FDR). We used R [39] for these analyses as well.

## 3. Results

### 3.1. Descriptive Statistics

The dataset consisted of 4316 cats. Almost half of them (49%) were females, and the majority (69%) of the cats were neutered. The median age was 4.9 years, the youngest cat was 0.3 years old and the oldest was 22.7 years old. The age of 123 cats remained unknown. Cats were grouped into 26 breed groups: the number of cats within breed groups ranged from 44 (Turkish Van) to 1012 (landrace cat shorthair) (Supplementary Table S2). This dataset was collected between March 2019 and September 2020.

The test–retest dataset included 127 cats, and the time between the first and second answers varied from 36 to 106 days (mean = 66 days). The inter-rater dataset included 41 cats, and the time between the answers varied from 47 to 164 days (mean = 109 days). Three inter-raters had known the cat for 3 months to 1 year, 27 inter-raters for 1–5 years and 11 inter-raters for more than 5 years. Test–retest data were collected from January to November 2020, and inter-rater data were collected from August 2020 to January 2021.

### 3.2. Factor Structure

The factor structure with seven factors was the most coherent. We named the extracted factors as fearfulness, activity/playfulness, aggression toward humans, sociability toward humans, sociability toward cats, excessive grooming and litterbox issues (Table 1). This factor structure accounted for 43% of the variance in behavior and personality.

### 3.3. Reliability and Internal Consistency

The internal consistency of most factors was acceptable. Cronbach’s alpha varied from 0.60 (litterbox issues) to 0.90 (fearfulness), and Guttmann’s lambda 6 varied from 0.60 (excessive grooming) to 0.93 (fearfulness) (Table 2).

The test–retest reliability of all factors was good (Table 2). Test–retest reliabilities varied from 0.69 (excessive grooming) to 0.92 (aggression toward humans). The mean test–retest reliability of all factors was 0.83. Test–retest reliability estimates for individual items can be found in Supplementary Table S3.

Inter-rater reliabilities of all factors were good (Table 2). Aggression toward humans had the lowest ((ICC(1,1) = 0.61, ICC(1,k) = 0.75) and excessive grooming the highest inter-rater reliability (ICC(1,1) = 0.87, ICC(1,k) = 0.93). The mean inter-rater reliability of all factors was 0.83. Inter-rater reliabilities for individual items are shown in Supplementary Table S3.

### 3.4. Convergent and Discriminant Validity

We formed 25 hypotheses to validate the extracted factors, where 22 of these were met, and only 3 were not (Table 3). Two of the rejected hypotheses considered the factor litterbox issues, and one considered sociability toward humans.

We assessed the discriminant validity by evaluating the factor correlations (Table 4) and found only one moderate correlation (>0.30) occurring between sociability toward cats and aggression toward humans.

### 3.5. Breed Differences

**Figure 2:**
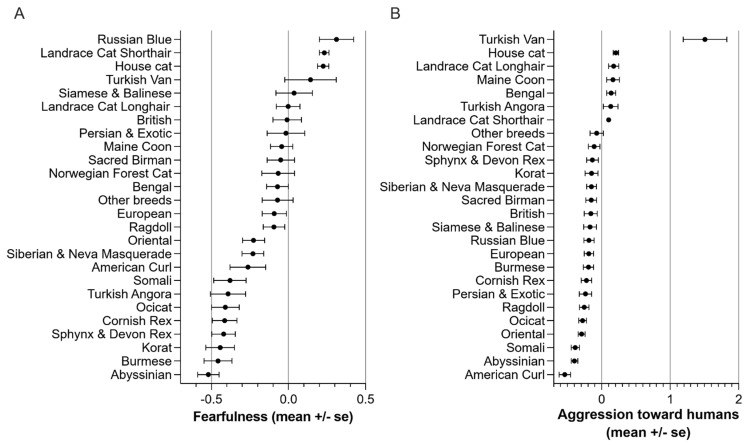
Breed differences in personality factors (**A**) fearfulness and (**B**) aggression toward humans.

When comparing breed mean scores, Kruskal–Wallis tests were statistically significant in all seven traits, meaning that at least some breeds differ from each other (Table 5). In fearfulness, Russian Blue scored the highest and Abyssinian the lowest (Figure 2A). In aggression toward humans, Turkish Van scored the highest and American Curl the lowest (Figure 2B).

**Figure 3:**
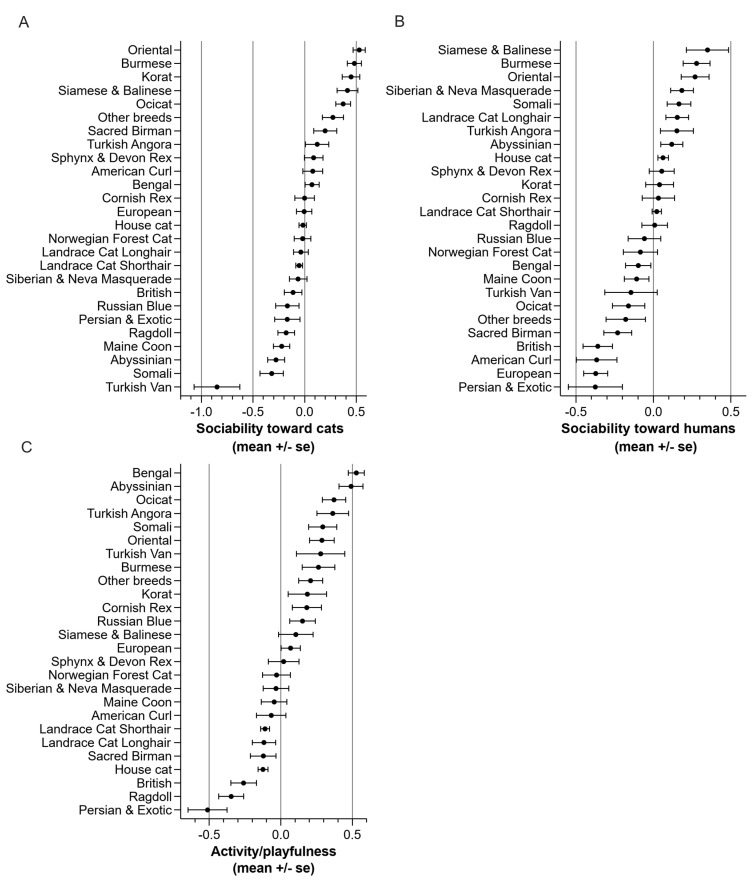
Breed differences in factors (**A**) sociability toward cats, (**B**) sociability toward humans and (**C**) activity/playfulness.

In sociability toward cats, Oriental scored the highest and Turkish Van the lowest (Figure 3A). In sociability toward humans, Siamese and Balinese had the highest score and Persian and Exotic the lowest (Figure 3B). In activity/playfulness, Bengal had the highest score and Persian and Exotic the lowest (Figure 3C).

**Figure 4:**
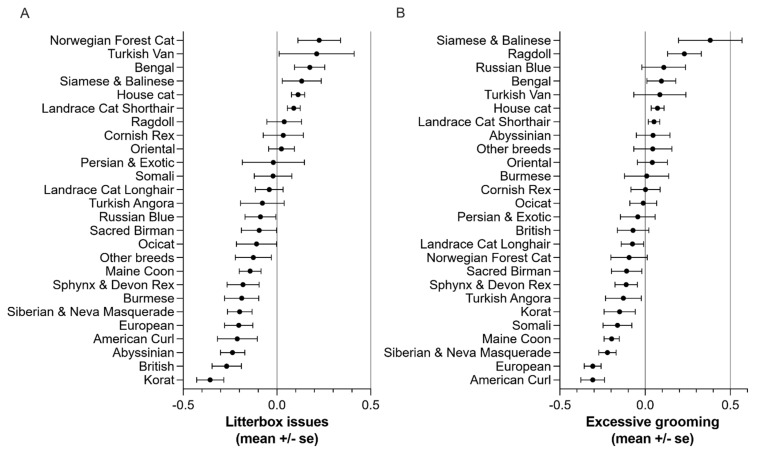
Breed differences in factors (**A**) litterbox issues and (**B**) excessive grooming.

In litterbox issues, Norwegian Forest cat had the highest score and Korat the lowest (Figure 4A). Finally, in excessive grooming, Siamese and Balinese scored the highest and American Curl the lowest (Figure 4B).

## 4. Discussion

We studied feline behavior and personality with an owner-completed questionnaire. We inspected the structure of personality, and the reliability and validity of the questionnaire. Reliability in time and between observers, and the questionnaire’s convergent and discriminant validity and internal consistency were good. In addition, we examined breed differences in the factors that were found and discovered them in all factors.

Feline personality and behavior structure included seven factors: fearfulness, activity/playfulness, aggression toward humans, sociability toward humans, sociability toward cats, excessive grooming and litterbox issues. The obtained factor structure is not the same as in previous feline personality and behavior studies, but the extracted factors share similarities with prior studies [2,15,17,18,19,22,23,37]. The structure of our questionnaire was similar to the structure of Fe-BARQ [19], but we used different methods to evaluate the optimal number of factors. Thus, compared to our 7 extracted factors, other studies extracted many more, some with 23 factors.

Fearfulness paralleled factors previously named shyness [31,37] and neuroticism [15,22,23]. The boldness–shyness axis is one of the best known animal personality traits [41]. Compared to Fe-BARQ, our fearfulness factor was mainly a combination of factors sociability, stranger-directed aggression and fear of novelty [19]. Similarly, our activity/playfulness factor was familiar from the previous literature [18,37] and resembled factors previously named extraversion [15] and openness [22,23]. In Fe-BARQ, items in this factor are divided into three factors: playfulness/activity, predatory behavior and prey interest [19].

Our aggression toward humans factor was a combination of factors touch sensitivity/owner-directed aggression and resistance to restraint from Fe-BARQ [19], sometimes labeled as roughness [22,23] and dominance [15]. On the other hand, our sociability toward humans factor resembled a combination of sociability, directed calls/vocalizations, purring, attention seeking, separation-related behavior and trainability factors from Fe-BARQ [19]. Similar traits have been labeled as contact to people [31,37], human nonsocial [17], friendliness [22,23] and amiability [18]. Our sociability toward cats factor was similar to factors named cat social [17], aggression towards other cats [31,37], familiar cat aggression [19] and dominance [15].

The last two factors, excessive grooming and litterbox issues, were behaviors rather than personality traits, but scoring high in these factors can suggest that those individuals are less stress-tolerant or have a disposition to react actively to stressful situations. The excessive grooming factor also existed in Fe-BARQ, and this problem has previously been studied in the Finnish cat population [31,37]. In Fe-BARQ, litterbox issues are divided into two factors, inappropriate elimination and elimination preferences.

### 4.1. Validity and Reliability

The internal consistency of most factors was acceptable, ranging from 0.60 (litterbox issues) to 0.90 (fearfulness), with a mean of 0.77. Most factors surpassed the suggested cut-off value of 0.70 for Cronbach’s alpha [42,43]. However, for sociability toward humans, Cronbach’s alpha was exactly 0.70, and for two other factors, the values were less than that, 0.66 for excessive grooming and 0.60 for litterbox issues. Excessive self-grooming included only three items and litterbox issues only six items, which may explain the low internal consistency, as Cronbach’s alpha is strongly affected by the length of the scale [44]. In addition, they are rare events that the majority of the cats never encounter [34], and thus answers in loading items lacked variability. In previous feline behavior and personality studies, Cronbach’s alpha values ranged between 0.50 and 0.94 [15,18,19,22,23].

Inter-rater values for the final factors were good, as all factors exceeded the preferable cut-off value of 0.60 [45]. ICC(1,1) ranged between 0.61 (aggression toward humans) and 0.87 (excessive grooming), with a mean of 0.72. ICC(1,k) ranged between 0.75 (aggression toward humans) and 0.93 (excessive grooming), with a mean of 0.83. In a previous feline personality study, factors’ inter-rater reliabilities ICC(3,1) varied between 0.31 and 0.55, and ICC(3,k) varied between 0.58 and 0.79 [12]. In the final factors, our item-level inter-rater reliability varied between 0.10 and 0.97 (ICC(1,1)) and 0.18 and 0.98 (ICC(1,k)). In previous feline personality studies, item-level inter-rater reliability varied between 0.07 and 0.91 [13,21], but these studies used a different estimation method.

The test–retest reliability was good, ranging from 0.69 (excessive grooming) to 0.92 (aggression toward humans). Correlations that exceed 0.70 can be considered good [43], and only excessive grooming slightly failed to reach this value. The mean test–retest reliability estimate for the questionnaire was 0.83. At the item level, correlations between time points varied between 0.35 and 0.93. In one previous feline study, factors’ test–retest reliability estimates varied between 0.63 and 0.76 [23]. To our knowledge, no other survey studies have reported test–retest estimates.

We examined the convergent validity of the questionnaire with 25 hypotheses, which were mainly based on the previous literature. Our questionnaire data agreed with most hypotheses, but three were not met; two of them addressed litterbox issues and one sociability toward humans. We hypothesized that male cats would have more litterbox issues than female cats and that cats living in multicat households would have more litterbox issues than cats living alone, but these hypotheses did not hold in our data. The previous literature supporting these hypotheses studied inappropriate elimination [32], urine marking [10] or inappropriate urinating [33,36], but our litterbox issues factor included substrate preference and cleanliness aspects as well. In contrast, Barcelos and colleagues [36] did not find that sex, litterbox cleanliness or substrate type would affect urinating or marking behavior. Instead, they noticed that cats in multicat households had more marking and urination problems than cats living alone, but we could not replicate this association in our dataset. In addition, we hypothesized that cats with owner-reported problematic behavior would have lower sociability toward humans than cats without reported behavior problems. After FDR correction, this relationship was not statistically significant (*p*-value = 0.052). Again, our sociability toward humans factor included more aspects than the sociability factor in the study of Duffy and colleagues [19]. Their concept included mainly social attitudes towards unfamiliar people, but our factor included, for example, purring and attention seeking from the owner as well. These hypotheses were only studied to evaluate the convergent validity of the questionnaire and factors. Therefore, in the future, the differences between, for example, male and female cats should be explored further.

Lastly, we examined the discriminant validity of the questionnaire by investigating the correlations between factors. We found only one moderate, negative correlation between aggression toward humans and sociability toward cats. This was a surprising and novel finding, as it has not been previously reported. However, our sociability toward cats factor also included items related to cat aggression, and in our previous study [37], we found a correlation between aggression toward humans and cats in the Finnish cat population. The correlation was phenotypic, but in Maine Coon, the traits were genetically correlated as well. Other correlations between factors were small, which indicates good discriminant validity.

### 4.2. Breed Differences

In addition to questionnaire validation and reliability, we briefly examined the possible association of breed with behavior and personality factors. In all seven factors, Kruskal–Wallis tests were significant, meaning that at least some of the breeds and breed groups differ from each other. This was expected, as differences in personality and behavior between cat breeds have been identified in multiple studies [10,19,35,37,46,47]. Interestingly, the order of the breeds was remarkably similar to the earlier studies which used a completely different study approach, namely, ratings by veterinarians [10,11]. Breed differences were also found in a previous study from the Finnish cat population, and the order of the breeds was quite similar in one parallel factor, activity/playfulness [37]. The most active and playful breeds were Bengal and Abyssinian, and the least active were Persian and Exotic, Ragdoll and British. This also replicates the results of two other previous studies [11,19]. Similarly, Bengals showed the most predatory behavior and Persians the least in the study of Wilhelmy and colleagues, in which they used Fe-BARQ [47]. This may indicate that, at least, Bengals have very high activity and prey interest, and Persians tend to score low in different cat populations.

In this study, the most fearful breeds were Russian Blue, landrace cat shorthair and house cat, and the boldest Abyssinian, Burmese and Korat. In our previous study [37], Russian Blues, house cats and Bengals showed the highest probability of shyness toward strangers. Similarly, Russian Blue was one of the breeds showing nervousness in the study of Takeuchi and Mori, but so did Abyssinian, which was among the least fearful breeds in our study [11]. Interestingly, long- and short-haired landrace cats seemed to differ from each other in fearfulness, with short-haired cats higher in the rank order. In aggression toward humans, the most aggressive breeds, Turkish Van and house cat, also scored high in aggression toward family members and strangers in our previous study [37]. In addition, low aggressiveness scores were obtained in both studies by Abyssinians, Somalis and Orientals. However, the British breed was closer to the average score in our study, whereas it was the least aggressive in our previous study [37]. In previous studies, Maine Coon [11,19,47] and Burmese [19] have been ranked among the breeds showing the most touch sensitivity/owner-directed aggression, and they also scored high in our study.

In sociability toward cats, the most social breeds were Oriental (Shorthair and Longhair), Burmese and Korat, and the least social was Turkish Van. Turkish Van had the highest probability of aggression toward other cats in our previous study [37], but the order of the other breeds differed. Unfortunately, personality studies from other countries’ Turkish Van populations do not exist. Thus, we cannot say whether this is a typical phenomenon only in the Finnish population. Interestingly, Oriental was more likely to show cat aggression than other breeds in the study of Wilhelmy and colleagues [47]. The number of Orientals in their study, however, was small (N = 16). On the other hand, Abyssinians and Siameses scored high in aggression to cats in another study [11]. Our sociability toward humans factor resembled reversed decreased contact from our previous study [37]. In our study, the most human social breeds were Siamese and Balinese, Burmese and Oriental, and the least social breeds were Persian and Exotic, European, American Curl and British. In our previous study [37], Oriental breeds (Siamese, Balinese, Oriental Shorthair and Longhair) had the lowest probability of decreased contact, and British, Sacred Birman, European and Persians the highest, paralleling our results. Persians scored the lowest in affection demand in the study of Takeuchi and Mori as well [11]. Burmese, Ragdoll and Maine Coon were ranked the most attention-seeking breeds [19], but only Burmese was replicated in our sociability toward humans factor. However, Siamese was ranked the second in the sociability factor, which joins results between studies. Conversely, Wilhelmy and colleagues reported that Siamese was less likely to show sociability toward humans than other breeds [47].

The breeds exhibiting the most excessive grooming were Siamese and Balinese, and Ragdoll. Oriental breeds also showed a high probability of excessive grooming in our previous study examining the Finnish cat population [37]. The order of the other breeds was not replicated; however, in this previous study, we also included other variables in their analysis than the breed of the cat. Interestingly, as we were able to inspect Oriental Shorthairs and Longhairs, and Siameses and Balineses as separate groups, we found that excessive grooming might be a problem in Siameses and Balineses, but not so much in Orientals. Wilhelmy and colleagues did not report breed differences in excessive grooming but noticed that Orientals more likely showed other compulsive behaviors [47]. In our study, the breeds having the most inappropriate elimination were Norwegian Forest cat, Turkish Van and Bengal, although the differences between breeds were minor. Bengal also showed inappropriate elimination more likely than other breeds in a previous study [47]. Further, we did not find Persian to be more prone to inappropriate elimination than other breeds, as some previous studies suggest [35,46]. In addition, the order of breeds greatly differed from the order in Takeuchi and Mori [11]. However, our litterbox issues factor included substrate preference and cleanliness aspects as well, which may explain these differences.

### 4.3. Limitations of This Study

This study has several limitations. We could not include items handling dogs and unfamiliar cats/kittens, as these items had high missingness. These behaviors would be interesting to study, for example, with a smaller subset of the data. Owners may have reported the breed, age or sex of their cat incorrectly, and especially for non-pedigree cats, this information cannot be confirmed. In addition, owners can have breed-specific expectations that may influence the results, for example, in activity/playfulness. It is also possible that some cats were already deceased a long time ago, but owners failed to report this. The study population may not reflect the whole cat population of Finland, as not all people are eager or able to participate in internet-based surveys. We advertised this study mostly on social media, and not all cat owners are active on that platform. In addition, although both excessive grooming and inappropriate elimination formed factors, they are not personality traits, and it would be more accurate to study them further based on the real answers in the questionnaire, not the factor scores. The factor structure of these factors also varied at different time points of statistical analysis, suggesting that the structure is not stable. Further, in this manuscript, we did not study pairwise differences between breeds, and thus we cannot state that certain breeds differ from each other. In addition, the possible effect of neutering was not studied. We will study these personality traits and breed differences with more complex analyses in the following manuscripts.

## 5. Conclusions

This study examined the structure, test–retest reliability, inter-rater reliability, convergent validity and discriminant validity of a feline behavior and personality questionnaire and briefly examined the breed differences in personality and behavior. The questionnaire included five personality and two problematic behavior-related factors, which appeared reliable and valid. Breeds differed in all traits, partly suggesting a genetic background for behavior and personality differences.

## Figures and Tables

**Table 1:** Item loadings in the feline behavior and personality questionnaire. Item names are abbreviated from the original statements, and the whole statements can be seen in Supplementary Table S3.

N.	Item (Abbreviated)	Fearfulness	Activity/ Playfulness	Aggression Toward Humans	Sociability Toward Humans	Sociability Toward Cats	Excessive Grooming	Litterbox Issues
72	Growls/hisses (unfam. adults)	**0.36**	0.11	**0.34**	−0.10	−0.23	0.03	0.04
90	Growls/hisses petting (unfam. person)	**0.35**	0.09	**0.45**	−0.11	−0.22	0.00	0.01
125	Suspicious	**0.84**	−0.02	−0.10	0.01	−0.03	−0.03	−0.02
137	Escapes/hides (unfam. people)	**0.94**	−0.02	−0.04	0.10	0.05	−0.03	0.01
117	Freezes (at vet)	**0.53**	−0.07	0.03	0.11	0.08	0.09	0.06
133	Hides/escapes (fam. prepares to leave)	**0.38**	−0.05	0.10	0.11	0.01	0.08	0.08
78	Runs/hides noise outside	**0.44**	0.01	0.09	0.17	0.13	0.03	0.09
65	Restless/fearful (home modified)	**0.41**	−0.03	0.11	0.18	0.06	0.12	0.26
36	Restless/fearful (unfam. objects)	**0.48**	−0.04	0.09	0.14	0.09	0.20	0.19
29	Stares (unfam. people)	**0.32**	0.14	0.06	0.09	0.00	0.00	0.05
113	Easily scared	**0.62**	−0.04	0.06	0.17	0.12	0.11	0.07
92	Runs/hides noise inside	**0.64**	−0.06	0.07	0.22	0.17	0.05	0.05
31	Greets unfam. adults	**−0.85**	−0.04	0.04	0.20	0.07	0.04	0.02
66	Greets unfam. children	**−0.78**	−0.02	−0.02	0.09	0.12	0.03	0.05
35	Comfortable/relaxed in social gatherings	**−0.90**	0.02	0.03	−0.02	0.00	0.01	0.01
58	Comfortable/relaxed petted (unfam. people)	**−0.83**	−0.09	−0.03	0.22	0.07	0.01	0.04
27	Comfortable/confident (unfam. places)	**−0.65**	0.09	−0.09	−0.09	−0.04	−0.07	−0.01
56	Confident at home	**−0.45**	0.20	0.01	0.13	0.03	−0.14	−0.13
55	Comfortable playing (unfam. people)	**−0.82**	0.05	−0.03	0.09	0.10	0.01	0.00
103	Active	−0.09	**0.62**	−0.02	−0.06	0.11	−0.06	−0.04
96	Waiting fam. member at door	−0.16	**0.34**	−0.02	0.28	−0.02	0.01	−0.05
122	Carries toys	0.00	**0.59**	−0.06	0.00	0.07	0.06	−0.04
132	Chases shadows/lights	0.00	**0.46**	0.01	−0.04	0.06	−0.01	−0.06
12	Chases imaginary objects	0.08	**0.33**	0.09	0.07	0.06	0.17	0.05
99	Chases small animals	0.03	**0.43**	0.02	−0.09	−0.09	−0.18	−0.01
109	Enjoys jumping/climbing	−0.01	**0.58**	−0.05	−0.11	−0.03	−0.06	0.05
111	Gets excited (animals at the window)	0.06	**0.51**	0.02	0.02	−0.07	−0.10	0.01
24	Gets excited (new toys)	−0.05	**0.55**	−0.05	−0.03	0.17	−0.06	−0.03
70	Interactive play (people)	0.00	**0.58**	−0.08	0.11	0.00	0.05	−0.03
43	Is persevering	−0.16	**0.32**	0.24	0.08	0.00	0.03	0.01
71	Moves elegantly	−0.02	**0.34**	−0.06	−0.09	−0.06	−0.14	0.01
112	Sudden bursts of running	0.04	**0.50**	0.03	0.10	0.18	0.06	−0.02
91	Plays fetch	−0.03	**0.53**	−0.04	0.08	0.00	0.03	−0.04
64	Finds new ways to get attention	−0.13	**0.50**	0.01	0.25	−0.02	0.05	0.04
28	Associate things	−0.09	**0.38**	−0.04	0.15	−0.04	−0.09	0.02
44	Reacts to video/sound (TV/computer)	0.04	**0.41**	−0.06	0.03	0.05	0.05	−0.02
52	Rests/sleeps in elevated places	0.04	**0.39**	0.01	−0.13	−0.01	−0.04	0.09
86	Runs around playing	0.02	**0.62**	−0.01	0.04	0.16	−0.02	−0.06
105	Stalks moving objects	0.01	**0.66**	−0.05	−0.02	0.10	−0.07	−0.07
77	Walks to front door (fam. member leaving)	−0.16	**0.35**	0.02	0.25	−0.03	0.06	−0.01
110	Chases/ambushes fam. members	−0.02	**0.44**	0.07	0.00	0.26	0.03	0.03
80	Scratch/bite fam. cat (fam. cat staring/growling/hissing)	−0.01	0.07	**0.31**	0.02	**−0.38**	0.08	0.14
138	Scratch/bite (petted on the belly)	0.03	0.01	**0.66**	−0.03	−0.05	−0.02	0.00
53	Scratch/bite (brushed)	0.00	0.00	**0.67**	0.02	−0.01	−0.08	0.00
75	Scratch/bite (vet)	−0.01	−0.02	**0.74**	0.03	0.00	0.00	−0.02
30	Scratch/bite (nails clipped)	−0.06	−0.02	**0.87**	0.02	0.05	0.00	−0.01
38	Scratch/bite (petted base of the tail)	−0.04	0.00	**0.67**	−0.01	−0.01	0.04	0.01
23	Chases/bites moving legs/feet	−0.04	0.22	**0.37**	0.01	0.04	0.00	0.08
6	Growls/hisses (approached by human while having food)	−0.04	0.15	**0.37**	−0.02	−0.10	−0.07	0.12
61	Growls/hisses (vet)	−0.01	0.03	**0.60**	0.00	−0.13	0.03	−0.02
46	Growls/hisses (medicine, fam. person)	0.03	−0.04	**0.80**	0.02	−0.01	−0.02	−0.02
128	Growls/hisses (nails clipped)	−0.04	−0.01	**0.79**	−0.06	−0.05	−0.03	−0.05
73	Growls/hisses (petted base of the tail)	−0.02	−0.05	**0.72**	−0.07	−0.07	0.03	0.02
114	Unexpectedly scratches/bites (petted)	−0.03	0.11	**0.61**	−0.02	−0.06	0.04	0.02
5	Scratch/bite (medicine, fam. person)	0.03	−0.10	**0.84**	0.02	0.10	−0.01	0.01
59	Squirms/escape (picked up/held)	0.19	0.05	**0.30**	−0.26	0.06	0.08	0.03
104	Human-oriented	−0.13	0.03	−0.06	**0.47**	**−0.58**	−0.01	−0.06
120	Squirms/escape (in lap)	0.24	0.10	0.23	**−0.32**	0.00	0.06	0.02
26	“Talks” to people	−0.01	0.22	−0.02	**0.49**	−0.02	−0.02	0.02
8	Always purrs when petted	−0.03	−0.08	−0.10	**0.55**	0.01	−0.12	−0.02
39	Comes when called	−0.09	0.17	−0.10	**0.38**	−0.05	−0.02	−0.01
37	Seeks physical contact (people)	−0.21	0.05	−0.12	**0.60**	0.01	−0.02	−0.06
21	Purrs (in lap)	−0.02	−0.02	−0.04	**0.54**	−0.05	−0.09	−0.06
54	Reacts by vocalizing	0.07	0.27	0.04	**0.39**	0.00	0.04	0.05
127	Restless (fam. people shows affection to person)	0.02	0.20	0.09	**0.32**	−0.03	0.19	0.13
115	Restless (fam. people shows affection pet)	0.04	0.18	0.15	**0.35**	−0.02	0.12	0.11
101	Restless/pacing (fam. member leave)	0.13	0.23	0.08	**0.38**	−0.01	0.16	0.08
57	Lie on things used	−0.09	0.22	0.03	**0.33**	−0.10	0.02	0.00
15	Vocalizes (left alone)	0.01	0.25	0.05	**0.34**	−0.03	0.17	0.09
118	Enjoys playing with cats	−0.02	0.27	−0.02	0.00	**0.75**	−0.04	−0.04
119	Seeks company (cats)	−0.06	0.11	0.03	0.01	**0.80**	−0.03	−0.01
17	Seeks physical contact (cats)	−0.06	0.10	0.00	−0.01	**0.77**	0.02	0.06
102	Gets along (cats)	−0.04	−0.04	−0.08	0.05	**0.71**	−0.11	−0.11
60	Growls/hisses (fam. cat staring/growling/hissing)	0.02	0.08	0.20	0.04	**−0.54**	0.04	0.14
67	Growls/hisses in favorite resting place (fam. cat)	0.01	0.04	0.22	−0.01	**−0.59**	0.05	0.15
76	Exhibits self-mutilation	−0.02	−0.03	−0.05	−0.05	−0.03	**0.85**	−0.03
51	Sudden frantic licking/chewing (self)	0.05	0.01	0.09	0.07	−0.02	**0.68**	−0.06
98	Excessive/intensive grooming	−0.04	−0.01	−0.06	−0.06	−0.01	**0.91**	0.00
124	Defecates inappropriate places	−0.05	−0.13	−0.02	−0.02	0.02	−0.02	**0.74**
13	Refuses shared litter box	0.00	0.06	0.01	−0.03	−0.22	0.03	**0.62**
48	Prefers some cat litters	0.02	0.11	0.01	0.07	0.04	0.05	**0.37**
88	Refuses dirty litter box	0.03	0.07	−0.01	0.07	0.00	−0.01	**0.63**
100	Sprays indoors	−0.07	0.04	−0.09	−0.15	−0.03	−0.08	**0.57**
4	Urinates inappropriate places	−0.01	−0.05	−0.05	−0.02	0.03	−0.03	**0.79**

Loading items (≥|0.3|) are in bold.

**Table 2:** Internal consistency, test–retest reliability and inter-rater reliability of feline behavior and personality factors. ICC = intraclass correlation coefficient.

	Internal Consistency		Test–Retest Reliability	Inter-Rater Reliability	
Factor	Cronbach’s Alpha	Guttman’s Lambda 6	Correlation	ICC(1,1)	ICC(1,*k*)
Fearfulness	0.90	0.93	0.91	0.65	0.79
Activity/playfulness	0.84	0.87	0.89	0.65	0.78
Aggression toward humans	0.83	0.88	0.92	0.61	0.75
Sociability toward humans	0.70	0.76	0.82	0.71	0.83
Sociability toward cats	0.83	0.86	0.78	0.72	0.84
Excessive grooming	0.66	0.60	0.69	0.87	0.93
Litterbox issues	0.60	0.61	0.81	0.83	0.91
Mean	0.77	0.79	0.83	0.72	0.83

**Table 3:** Hypotheses, their Pearson correlation coefficients, *t*-test statistics, sample sizes and *p*-values used to examine the questionnaire’s convergent validity. All *p*-values were corrected for the false discovery rate (FDR).

Factor	Hypothesis	Test	Statistic	*n*	*p*-Value
Fearfulness	Female cats more fearful	*t*-test	4.95, df = 4254.7	4316	**<0.0001**
	Russian Blue, house cat, Bengal and European more fearful than Cornish Rex, Burmese, Persian and Exotic	*t*-test	−7.63, df = 346.02	1421	**<0.0001**
	Cats with owner-reported problematic behavior more fearful	*t*-test	−5.12, df = 431.62	2184	**<0.0001**
Activity/ playfulness	Older cats less active/playful	correlation	−0.43	4193	**<0.0001**
	Fearful cats less active/playful	correlation	−0.15	4316	**<0.0001**
	Cornish Rex, Korat, Bengal and Abyssinian more active/playful than British, Ragdoll, Sacred Birman, Siberian, Neva Masquerade, Persian and Exotic	*t*-test	10.32, df = 954.84	972	**<0.0001**
Aggression toward humans	Older cats more aggressive	correlation	0.11	4193	**<0.0001**
	Cats living in multicat households less aggressive	*t*-test	4.81, df = 1067.9	3319	**<0.0001**
	Turkish Van and house cat more aggressive than British, Persian, Exotic and Oriental breeds*	*t*-test	8.61, df = 1011.5	1216	**<0.0001**
Sociability toward humans	Female cats less sociable	*t*-test	−7.25, df = 4221.4	4316	**<0.0001**
	Fearful cats less sociable	correlation	−0.24	4316	**<0.0001**
	Active/playful cats more sociable	correlation	0.21	4316	**<0.0001**
	Korat, Oriental breeds* and Abyssinian more social than British, Sacred Birman, European Persian and Exotic	*t*-test	−6.51, df = 516.9	632	**<0.0001**
	Cats with owner-reported problematic behavior less social *Not met*	*t*-test	1.67, df = 407.87	2184	0.052
Sociability toward cats	Older cats less sociable	correlation	−0.34	4193	**<0.0001**
	Female cats less sociable	*t*-test	−11.70, df = 3909.3	4316	**<0.0001**
	Fearful cats less sociable	correlation	−0.12	4316	**<0.0001**
	Cats with owner-reported problematic behavior less sociable	*t*-test	4.89, df = 397.33	2184	**<0.0001**
Excessive grooming	Fearful cats have more excessive grooming	correlation	0.16	4316	**<0.0001**
	Burmese and Oriental breeds* have more excessive grooming than Siberian, Neva Masquerade and Norwegian Forest cat	*t*-test	3.25, df = 474.91	502	**<0.001**
	Cats with owner-reported problematic behavior have more excessive grooming	*t*-test	−6.91, df = 364.49	2184	**<0.0001**
Litterbox issues	Older cats have more litterbox issues	correlation	0.15	4193	**<0.0001**
	Male cats have more litterbox issues *Not met*	*t*-test	0.09, df = 4254.80	4316	0.484
	Cats living in multicat households have more litterbox issues *Not met*	*t*-test	1.21, df = 1315	3319	0.888
	Cats with owner-reported problematic behavior have more litterbox issues	*t*-test	−11.59, df = 352.38	2184	**<0.0001**

* Oriental breeds = breed groups “Oriental” and “Siamese and Balinese”. Statistically significant *p*-values (<0.05) are in bold.

**Table 4:** Factor correlations in the feline behavior and personality questionnaire.

Factor	Fearfulness	Activity/ Playfulness	Human Aggression	Human Sociability	Cat Sociability	Excessive Grooming	Litterbox Issues
Fearfulness	1	−0.15	0.27	−0.24	−0.13	0.15	0.18
Activity/ playfulness	−0.15	1	0	0.21	0.29	−0.07	−0.09
Aggression toward humans	0.27	0	1	−0.10	**−0.31**	0.21	0.21
Sociability toward humans	−0.24	0.21	−0.10	1	0.04	0.11	0.05
Sociability toward cats	−0.13	0.29	**−0.31**	0.04	1	−0.16	−0.20
Excessive grooming	0.15	−0.07	0.21	0.11	−0.16	1	0.22
Litterbox issues	0.18	−0.09	0.21	0.05	−0.20	0.22	1

Moderate correlations are in bold.

**Table 5:** Breed differences in feline personality and behavior factors. All *p*-values were corrected for the false discovery rate (FDR). *n* = 4316, df = 25.

Factor	χ^2^	*p*-Value
Fearfulness	291.85	<0.0001
Activity/playfulness	232.23	<0.0001
Aggression toward humans	324.94	<0.0001
Sociability toward humans	114.27	<0.0001
Sociability toward cats	216.38	<0.0001
Excessive grooming	104.73	<0.0001
Litterbox issues	124.67	<0.0001

## Data Availability

The data presented in this study are openly available in FigShare: https://figshare.com/account/articles/14899077 (accessed on 2 July 2021).

## Supplementary Materials

The following are available online at https://www.mdpi.com/article/10.3390/ani11071991/s1, Table S1. Hypotheses and their references used to validate the cat behavior questionnaire, Table S2. Breeds and breed groups with basic demographics in the questionnaire, Table S3. Item-level missingness, test–retest reliabilities and inter-rater reliabilities in the questionnaire, Supplementary File: Feline personality and behavior questionnaire.
