# Supplementary material for: Reliability and Validity of Seven Feline Behavior and Personality Traits
Source: Animals (Basel). 2021 Jul 2;11(7):1991. doi: 10.3390/ani11071991 (PMC8300181; doi:10.3390/ani11071991)
Supplement: Supplementary file 1 [file animals-11-01991-s001.zip › animals-1268662-supplementary.pdf]

Reliability and validity of seven feline behavior and personality traits

Salla Mikkola, MSc[1,2,3], Milla Salonen, PhD[1,2,3], Emma Hakanen, MSc[1,2,3], Sini Sulkama, MSc[1,2,3], Hannes Lohi, PhD, prof.[1,2,3*]

[1]Department of Veterinary Biosciences, University of Helsinki, Helsinki, Finland

[2]Department of Medical and Clinical Genetics, University of Helsinki, Helsinki, Finland

[3]Folkhälsan Research Center, Helsinki, Finland

* Corresponding author:

Hannes Lohi, PhD, Professor

+358294125085

Email: hannes.lohi@helsinki.fi

PL 63 (Haartmaninkatu 8), 00014 HELSINGIN YLIOPISTO, Finland

Supplementary information

Table S1. Hypothesis and their references used to validate the cat behavior questionnaire.

Factor	Hypothesis	References
Fearfulness	Female cats more fearful	[1,2]
	Russian Blue, House cat, Bengal and European more fearful than Cornish Rex, Burmese, Persian and Exotic	[3]
	Cats with owner-reported problematic behavior more fearful	[1,4]
Activity/playfulness	Older cats less active/playful	[4,5]
	Fearful cats less active/playful	[5,6]
	Cornish Rex, Korat, Bengal and Abyssinian more active/playful than British, Ragdoll, Sacred Birman, Siberian, Neva Masquerade, Persian and Exotic	[3]
Aggression toward humans	Older cats more aggressive	[7]*
	Cats living in multicat households less aggressive	[1,8,9]
	Turkish Van and House cat more aggressive than British, Persian, Exotic and Oriental breeds*	[3,10]
Sociability toward humans	Female cats less sociable	[1]
	Fearful cats less sociable	[3]
	Active/playful cats more sociable	[3]
	Korat, Oriental breeds* and Abyssinian more social than British, Sacred Birman, European, Persian and Exotic	[3]
	Cats with owner-reported problematic behavior less social	[4]
Sociability toward cats	Older cats less social	[1]
	Female cats less sociable	[1,9,11]
	Fearful cats less sociable	[1]
	Cats with owner-reported problematic behavior less social	[4]
Excessive grooming	Fearful cats have more excessive self-grooming	[1]
	Burmese and Oriental breeds* have more excessive self-grooming than Siberian, Neva Masquerade and Norwegian Forest Cat	[3]
	Cats with owner-reported problematic behavior have more excessive self-grooming	[4]
Litterbox issues	Older cats have more litterbox issues	[4,12]
	Male cats have more litterbox issues *Not met*	[2,7,13]
	Cats living in multicat households have more litterbox issues *Not met*	[12]
	Cats with owner-reported problematic behavior have more litterbox issues	[4]

* In the study of Naderi and others, aggressiveness includes both aggressiveness toward human and cats.

Table S2. Breeds and breed groups with basic demographics in the questionnaire. Sh = Shorthair, Lh = Longhair.

				Sex			
		**Age (years)**		Female		Male	
**Breed / breedgroup**	***n***	Mean	Median	Intact %	Neutered %	Intact %	Neutered %
Abyssinian	116	5.4	4.3	21	23	28	28
American Curl (Sh & Lh)	82	5.5	4.5	21	21	28	30
Bengal (Sh & Lh)	158	5.4	3.6	21	28	19	32
British (Sh & Lh)	108	4.7	3.2	23	32	19	25
Burmese (European & American)	69	5.6	4.6	29	26	16	29
Cornish Rex	91	6.2	5.3	23	25	12	40
European	160	5.7	4.5	24	34	19	23
House Cat (House Cat Sh & Lh, Domestic Sh & Lh, Mixed breed and cats without breed)	807	6.6	6.2	12	37	13	38
Korat	58	6.6	5.8	22	29	16	33
Landrace Cat Longhair	175	5.6	4.6	13	42	9	36
Landrace Cat Shorthair	1012	6.4	5.8	8	39	12	42
Maine Coon	159	4.5	3.3	23	19	23	35
Norwegian Forest Cat	95	6.2	5.5	13	35	18	35
Ocicat	109	5.8	5.0	16	30	17	37
Oriental (Oriental Sh & Lh, and Snowshoe)	128	5.5	4.6	16	33	15	36
Persian and Exotic (Persian, Exotic and Himalayan)	53	6.6	6.0	28	11	25	36
Ragdoll	161	5.3	4.3	20	29	16	35
Russian Blue	83	4.4	3.3	20	28	14	37
Sacred Birman	93	5.6	4.8	17	34	10	39
Siamese and Balinese (Siamese, Balinese and Seychellois)	76	5.8	5.5	16	30	16	38
Siberian and Neva Masquerade	134	4.5	3.3	24	22	22	31
Somali	81	5.9	4.4	17	33	19	31
Sphynx and Devon Rex	117	5.1	4.0	25	29	16	30
Turkish Angora	61	4.1	3.1	26	23	20	31
Turkish Van	44	7.7	7.3	14	43	9	34
Other breed (breeds with < 21 individuals)	86	4.1	2.6	24	21	20	35
Total	4316	5.8	4.9	16	33	15	36

Table S3. Item level missingness, test-retest reliabilities and inter-rater reliabilities in the questionnaire.

N.	Item	Missingness %	Test-retest reliability	Inter-rater reliability	
			Correlation	ICC(1,1)	ICC(1,*k*)
1	Often seeks out physical contact from people, nudges or nuzzles	0.0	0.57	0.49	0.66
2	Opens doors and cabinets	0.2	0.67	0.60	0.75
3	Rests or sleeps in secure places (e.g. in laundry baskets or wardrobes)	0.0	0.61	0.43	0.60
4	Reacts to the video or sound coming from the TV or computer screen (e.g. nature documentaries, cat videos, mouse cursor)	0.8	0.65	0.36	0.53
5	Refuses to use the litter box if it is dirty	4.6	0.84	0.63	0.77
6	Freezes when examined or treated by a veterinarian	7.3	0.78	0.52	0.68
7	Seems confident at home	0.1	0.69	0.59	0.74
8	Is comfortable and relaxed among people in social gatherings (e.g. parties)	3.8	0.82	0.65	0.79
9	Is easily scared even by small unexpected things and sounds	0.0	0.70	0.60	0.75
10	Defecates in inappropriate places indoors	0.3	0.65	0.76	0.86
11	Unexpectedly scratches or bites when petted	0.3	0.78	0.32	0.48
12	Always greets unfamiliar children visiting your home in a friendly manner (e.g. sniffs, rubs, purrs, lies on the floor)	22.5	0.69	0.47	0.64
13	Enjoys tearing up cardboard and paper	0.9	0.60	0.42	0.59
14	Runs or hides when hearing a sudden or loud noise coming from inside the house (e.g. vacuum cleaner, dropped object, instrument, doorbell)	0.0	0.63	0.27	0.42
15	Runs or hides when hearing a sudden or loud noise coming from outside the house (e.g. jackhammer, fireworks)	1.7	0.67	0.55	0.71
16	Asks to be let in and out of rooms (e.g. walks toward a door and meows)	0.6	0.59	0.51	0.67
17	Freezes and stares intensely at nothing visible	1.4	0.51	0.45	0.62
18	Tries to wake up members of the household early in the morning	0.3	0.65	0.38	0.55
19	Often exhibits sudden bursts of running (“zoomies”)	0.0	0.71	0.60	0.75
20	"Talks" to people (e.g. meows when spoken to)	0.0	0.79	0.57	0.73
21	Shows increased activity in the early hours of morning around dawn/sunrise	2.8	0.57	0.45	0.62
22	Chews or destroys objects when left alone at home	0.7	0.53	0.33	0.50
23	Reacts strongly to catnip by, e.g. rubbing, rolling over or licking	7.4	0.78	0.60	0.75
24	Chases rodents, birds, reptiles, squirrels, rabbits, insects or other small animals, given the opportunity	18.1	0.39	0.61	0.76
25	Always purrs when petted	0.3	0.70	0.54	0.71
26	Attempts to scratch or bite when approached by human while in possession of prey, stolen or especially delicious food**	3.9	0.58	-0.06	-0.13
27	Appears comfortable (confident, relaxed) when playing with familiar children*	34.5	0.58	0.63	0.77

28	Growls or hisses at a familiar cat when the familiar cat is staring, growling, or hissing at her/him	15.1	0.52	0.63	0.77
29	Attempts to scratch or bite familiar dogs*	52.3	0.73	0.57	0.73
30	Growls or hisses when examined or treated by a veterinarian	7.8	0.79	0.38	0.55
31	Growls or hisses at unfamiliar dogs*	46.2	0.83	0.22	0.36
32	Tends to lie on newspapers, books, keyboards or other things that are being used by people	0.3	0.74	0.43	0.60
33	Gets excited about new toys	0.2	0.78	0.36	0.53
34	Runs around in the house while playing	0.1	0.69	0.49	0.65
35	Greets unfamiliar adult cats in a friendly manner (sniffs, touches noses)*	36.8	0.67	0.32	0.49
36	Growls or hisses at familiar dogs*	53.1	0.80	0.80	0.89
37	Shows restlessness or fearfulness when his/her home is modified (e.g. furniture moved from usual places, fabrics or sheets changed)	2.9	0.77	0.43	0.60
38	Chews or eats house plants or cut flowers when given the opportunity (this does not include cat grass given for the cat to eat)	7.6	0.75	0.55	0.71
39	Makes eye contact with familiar people, simultaneously squinting or slowly blinking its eyes**	3.2	0.55	-0.06	-0.12
40	Scratches claws on inappropriate objects or surfaces (e.g. couch, curtains, wallpaper) indoors	0.2	0.77	0.61	0.76
41	Attempts to scratch or bite when petted at the base of the tail	0.6	0.82	0.15	0.26
42	Walks to the hallway/next to front door when a household member prepares to leave the home	0.4	0.63	0.58	0.74
43	Does not want to use the same litter box than the other cats in the household. Choose "I don't know" if he/she is the only cat in the household	17.7	0.49	0.14	0.24
44	If the target of the cat’s aggression is inaccessible, the cat attempts to scratch or bite a “wrong” accessible target (e.g. the cat sees an unfamiliar cat through the window and bites a family member)**	14.5	0.38	-0.03	-0.06
45	Enjoys jumping and climbing to high places	0.0	0.80	0.55	0.71
46	Shows restlessness (e.g. vocalizes) when a member of the household shows affection to a person	7.0	0.35	0.31	0.47
47	Gets excited (e.g. makes chirping or chattering noises, or lashes its tail) when looking at birds or other small animals at the window	0.8	0.67	0.40	0.57
48	Attempts to scratch or bite (in a non-playful way) when petted on the belly	0.9	0.88	0.79	0.89
49	Carries small objects or toys in his/her mouth	0.1	0.74	0.65	0.78
50	Attends and listens closely to everything you say or do**	0.1	0.57	0.07	0.13
51	While playing, always chases and ambushes other household members (e.g. people, dogs, or cats) playfully	1.1	0.56	0.21	0.35
52	Growls or hisses when his/her nails are clipped	13.5	0.93	0.55	0.71
53	Growls or hisses when given medicine by a familiar person (this does not include vets)	9.1	0.67	0.75	0.85
54	Chases or hunts imaginary objects	2.6	0.60	0.34	0.50
55	Attempts to scratch or bite when being bathed*	31.0	0.69	0.72	0.84
56	Growls or hisses when being bathed*	31.5	0.40	0.72	0.84
57	Shows increased activity in the evening around dusk/sunset	1.8	0.39	0.48	0.65

	or late at night				
58	Runs or hides from unfamiliar cats*	34.6	0.63	0.68	0.81
59	Attempts to scratch or bite when being brushed	4.1	0.86	0.10	0.19
60	Reacts similarly in similar situations (it is possible to predict how your cat will react)	1.4	0.35	0.10	0.19
61	Stares intensely (wide-eyed, not blinking) at unfamiliar people	7.2	0.59	0.23	0.37
62	Exhibits self-mutilation, e.g. pulls hairs off with teeth, vigorously nibbles or bites his/her body parts	0.6	0.35	0.81	0.90
63	Curiously investigates and explores new objects or changes in his/her environment**	0.2	0.58	0.07	0.12
64	Growls or hisses when approached by a familiar cat while eating**	15.4	0.64	-0.03	-0.07
65	Attempts to scratch or bite unfamiliar dogs*	57.5	0.72	0.76	0.86
66	Shows restlessness or irritation when other cats in the family are having a fight*	40.2	0.69	0.58	0.74
67	Shows excessive and intensive grooming (inhibits other behaviors) throughout the day	1.2	0.55	0.87	0.93
68	Shows restlessness or fearfulness when unfamiliar objects are introduced into the home	0.8	0.54	0.18	0.31
69	Plays fetch with people (retrieves thrown objects or toys)	0.8	0.85	0.45	0.62
70	Gets along well with other cats in the household. Choose “I don't know” if he/she is the only cat in the household	18.0	0.69	0.56	0.72
71	Seeks company of other cats in the household. Choose "I don't know" if he/she is the only cat in the household	17.7	0.71	0.52	0.68
72	When a baby is crying, cat goes to check the baby*	86.3	0.83	0.81	0.89
73	Growls or hisses when being brushed**	5.0	0.80	-0.01	-0.03
74	Chases his/her own tail/rear end	0.5	0.69	0.72	0.84
75	Attempts to scratch or bite when examined or treated by a veterinarian	8.2	0.77	0.69	0.82
76	Rests or sleeps on top of warm appliances (e.g.TV, printer, fireplace, radiator)	1.2	0.70	0.56	0.71
77	Attempts to scratch or bite when his/her nails are clipped	12.7	0.70	0.52	0.68
78	Greets unfamiliar kittens in a friendly manner (sniffs, touches noses)*	63.5	0.81	0.44	0.61
79	Moves elegantly and gracefully	0.1	0.70	0.29	0.45
80	Attempts to scratch or bite when given medicine by a familiar person (this do not include vets)	10.4	0.47	0.59	0.74
81	Hides or escapes when a household member prepares to leave the home	0.3	0.54	0.57	0.73
82	Urinates (crouching position) in inappropriate places	0.4	0.78	0.97	0.98
83	Escapes or hides from unfamiliar people	0.3	0.70	0.61	0.76
84	Is persevering, persistent and determined. Does what he/she wants even if he/she is told not to	0.3	0.59	0.27	0.42
85	Stalks, chases or pounces on moving objects (e.g. string, balls, soft toys)	0.1	0.51	0.33	0.49
86	Vocalizes by crying or meowing when left alone (without people) at home	13.2	0.74	0.55	0.71
87	Sprays (standing position with tail raised vertically and vibrating) indoors (e.g. furniture, walls)	0.8	0.46	0.46	0.63
88	Readily adapts to changes in environment or daily routines	0.9	0.55	-0.07	-0.14

	(e.g. changes in schedules, rearrangement of objects and furniture)**				
89	Squirms and tries to escape when picked up and held in arms	0.4	0.83	0.76	0.86
90	Brings prey animals into the home. If your cat has not opportunity to do this, choose “I don't know”*	83.0	0.71	0.74	0.85
91	Enjoys playing with other cats. Choose "I don't know" if he/she does not have an opportunity to play with other cats	16.2	0.69	0.65	0.79
92	Runs or hides from unfamiliar dogs*	49.3	0.66	0.15	0.25
93	Always comes to the hallway/next to front door or is already waiting there when a household member arrives home	0.2	0.71	0.52	0.69
94	Exhibits sudden frantic licking or chewing his/her body	0.7	0.49	0.39	0.56
95	Comes when called	0.1	0.77	0.45	0.62
96	Escapes or freezes when growled at, hissed at or otherwise threatened by a familiar cat	24.1	0.62	0.40	0.57
97	Chews cables and electric wires	0.6	0.79	0.69	0.82
98	Reacts to different events and stimuli by vocalizing (e.g. meowing)	1.1	0.58	0.41	0.59
99	Attempts to scratch or bite a familiar cat when the familiar cat is staring, growling, or hissing at him/her	20.4	0.80	0.38	0.55
100	Growls or hisses when petted at the base of the tail	0.9	0.47	0.73	0.84
101	Chews or licks plastics	1.9	0.85	0.57	0.72
102	Growls or hisses when an unfamiliar person tries to touch or pet him/her	1.4	0.76	0.59	0.75
103	Growls or hisses at unfamiliar children*	26.2	0.37	0.86	0.92
104	Chases or follows shadows or lights (e.g. laser pointers, flashlights)	4.0	0.82	0.58	0.74
105	Attempts to escape the home or yard/garden, if given the opportunity	12.9	0.75	0.67	0.81
106	Is comfortable and relaxed when being petted by unfamiliar people	0.8	0.78	0.63	0.77
107	Always greets unfamiliar adults visiting your home in a friendly manner (e.g. sniffs, rubs, purrs, lies on the floor)	0.8	0.74	0.50	0.67
108	Active, does not spend long time idle	0.0	0.71	0.27	0.43
109	Prefers specific types of cat litter	18.8	0.64	0.58	0.74
110	Shows restlessness (e.g. vocalizes) when a member of the household shows affection to another pet	12.6	0.71	0.55	0.71
111	Seeks physical contact from other cats in the household, e.g. sleeps next to them or grooms them. Choose “I don't know" if he/she is the only cat in the household	18.1	0.79	0.51	0.68
112	Growls or hisses when approached by a familiar cat in a favorite resting place	16.7	0.72	0.49	0.66
113	Friendly to unfamiliar dogs*	52.2	0.77	0.39	0.57
114	Growls or hisses when approached by human while in possession of prey, stolen or especially delicious food	4.7	0.70	0.15	0.26
115	Quickly finds new ways to get attention	2.2	0.68	0.21	0.34
116	Quickly learns to associate separate things (e.g. opening the fridge means getting food)	0.7	0.64	0.55	0.71
117	Concentration is not disturbed by anything (e.g. other cats) during a training session. Choose "I don't know" if you do not train your cat*	67.0	0.44	0.80	0.89
118	Sucks on or chews soft fabrics or wool	1.7	0.48	0.70	0.83

119	Growls or hisses at unfamiliar adults	1.6	0.48	0.63	0.77
120	Purrs when sitting on someone’s lap (has jumped up voluntarily)	4.0	0.46	0.19	0.32
121	Chases or bites people's moving legs or feet (in a non-playful way)	0.3	0.53	0.51	0.68
122	Does not seem to recognize (e.g. hisses or escapes) a familiar cat if it smells odd (e.g. the familiar cat has visited a vet clinic). Choose "I don't know" if he/she is the only cat in the household	24.1	0.65	0.74	0.85
123	Kneads on soft toys, fabrics, and other objects	0.3	0.77	0.58	0.73
124	Squirms and tries to escape when picked up to the lap of a sitting/lying person	1.1	0.73	0.62	0.76
125	Shows substrate preference when urinating or defecating in inappropriate places (e.g. laundry, bedding, carpets, flower pots). Choose "I don't know" if he/she does not urinate or defecate in inappropriate places*	77.1	0.73	0.61	0.76
126	Initiates interactive play with people (e.g. brings toys, strings, or candy wrappers to play with)	0.6	0.66	0.52	0.68
127	Repeats the same meaningless movements (e.g. pacing back and forth next to a wall)	2.4	0.79	0.10	0.18
128	Appears comfortable (confident, relaxed) when playing with unfamiliar people	5.9	0.82	0.59	0.75
129	Walks tail held upright at home	0.5	0.84	0.31	0.48
130	Does not approach people visiting his/her home right away (he/she is suspicious; however, he/she might greet people later on)	0.6	0.79	0.42	0.60
131	Rests or sleeps in elevated places (e.g. shelves, bookcases, tops of wardrobes or cupboards)	0.1	0.61	0.19	0.32
132	Seems comfortable and confident in unfamiliar places outside the home (e.g. cat shows)	24.0	0.78	0.43	0.60
133	Readily responds to commands taught to him/her (e.g. sit, down, paw). Choose "I don't know" if you have not trained him/her*	65.8	0.78	0.78	0.88
134	Picky: only eats foods he/she likes and leaves other foods untouched	0.3	0.82	0.79	0.89
135	Asks for food when hungry (e.g. meows next to his/her bowl)	0.7	0.65	0.51	0.68
136	Shows restlessness or pacing when a household member prepares to leave the home	0.7	0.63	0.45	0.62
137	Is fascinated by the activities of other small pets (e.g. rodents, birds, reptiles, fish) in the home*	75.4	0.57	0.19	0.31
138	Human-oriented: Enjoys the company of people more than the company of other cats	9.3	0.71	0.20	0.33

* Excluded from the analysis due to high item level missingness
** Removed from the analysis due to low reliability

Supplementary References

1. Ahola, M.K.; Vapalahti, K.; Lohi, H. Early weaning increases aggression and stereotypic behaviour in cats. *Sci. Rep.* **2017**, *7*, 10412, doi:10.1038/s41598-017-11173-5.
2. Hart, B.L.; Hart, L.A. *Your Ideal Cat: Insights into Breed and Gender Differences in Cat Behaviour*; Purdue University Press: West Lafayette, Indiana, 2013; ISBN 9781557536488.
3. Salonen, M.; Vapalahti, K.; Tiira, K.; Mäki-Tanila, A.; Lohi, H. Breed differences of heritable behaviour traits in cats. *Sci. Rep.* **2019**, *9*, 7949, doi:10.1038/s41598-019-44324-x.
4. Duffy, D.L.; de Moura, R.T.D.; Serpell, J.A. Development and evaluation of the Fe-BARQ: A new survey instrument for measuring behavior in domestic cats (Felis s. catus). *Behav. Processes* **2017**, *141*, 329–341, doi:10.1016/j.beproc.2017.02.010.
5. Bennett, P.C.; Rutter, N.J.; Woodhead, J.K.; Howell, T.J. Assessment of domestic cat personality, as perceived by 416 owners, suggests six dimensions. *Behav. Processes* **2017**, *141*, 273–283, doi:10.1016/j.beproc.2017.02.020.
6. de Rivera, C.; Ley, J.; Milgram, B.; Landsberg, G. Development of a laboratory model to assess fear and anxiety in cats. *J. Feline Med. Surg.* **2017**, *19*, 586–593, doi:10.1177/1098612X16643121.
7. Naderi, Mohammad; Rafiei, S.M.; Sattari, Behnoosh; Ale-Davoudl, S.J.; Seif, A.A.; Bokaei, S. The first study on classification of Iranian domestic cats' behavior problems and their associated risk factors. *Glob. Vet.* **2011**, *6*, 339–345.
8. Amat, M.; de la Torre, J.L.R.; Fatjó, J.; Mariotti, V.M.; Van Wijk, S.; Manteca, X. Potential risk factors associated with feline behaviour problems. *Appl. Anim. Behav. Sci.* **2009**, *121*, 134–139, doi:10.1016/j.applanim.2009.09.012.
9. Yamada, R.; Kuze-Arata, S.; Kiyokawa, Y.; Takeuchi, Y. Prevalence of 17 feline behavioral problems and relevant factors of each behavior in Japan. *J. Vet. Med. Sci.* **2020**, *82*, 272–278, doi:10.1292/jvms.19-0519.
10. Tamimi, N.; Malmasi, A.; Talebi, A.; Tamimi, F.; Amini, A. A survey of feline behavioral problems in Tehran. *Vet. Res. forum* **2015**, *6*, 143–147.
11. Ha, D.; Ha, J. A subjective domestic cat (Felis silvestris catus) temperament assessment results in six independent dimensions. *Behav. Processes* **2017**, *141*, 351–356, doi:10.1016/j.beproc.2017.03.012.
12. Barcelos, A.M.; McPeake, K.; Affenzeller, N.; Mills, D.S. Common risk factors for urinary house soiling (periuria) in cats and its differentiation: The sensitivity and specificity of common diagnostic signs. *Front. Vet. Sci.* **2018**, *5*, 1–12, doi:10.3389/fvets.2018.00108.
13. Strickler, B.L.; Shull, E.A. An owner survey of toys, activities, and behavior problems in indoor cats. *J. Vet. Behav. Clin. Appl. Res.* **2013**, *9*, 207–214, doi:10.1016/j.jveb.2014.06.005.

Supplementary file: Feline personality and behavior questionnaire

Feline personality questionnaire

This is the behavior and personality questionnaire for all cats developed by the Feline Genetics Group lead by Professor Hannes Lohi. The aim of this questionnaire is to assess the behavior and variation of behavior in cats. The questionnaire includes three sections: background and living conditions, behavior, and health status. Cats of any age, breed, and temperament can participate in the study. We hope that house cat owners will also participate in the study! To enable us to use your answers in the research, please fill **all three sections.**

The identity of you and your cat as well as your participation in the study will remain confidential. Read the privacy policy of the Feline Genetics Group (only in Finnish). Based on the survey answers, some cats may be invited to voluntary behavior tests or clinical examinations.

You can fill the Health status survey again anytime and it will show in available surveys.

Behavior

The next statements describe the cat’s reactions in different situations. If we have not defined a certain place in the statement, choose the option that best fits your cat in a normal home environment. There are no right or wrong answers, so please answer truthfully. If you have not been in certain situations with your cat or do not know how your cat behaves in these situations, choose “I don’t know”.

Definitions of some terms used: “**Scratching**” means that the cat’s claws are exposed. “**Biting**” does not mean gentle nipping but a bite that leaves a mark.

We will show a report of your cat’s personality after we have obtained answers of around 1000 cats. If you answer “I don’t know” to a question, the report of that part of the questionnaire will be empty.

Asterisk indicates mandatory field.

Data protection *

□ Hereby, I accept that my personal information, the information of my cat and all of the data collected with this questionnaire I have provided is transferred to Feline Genetics research group at the University of Helsinki and used in scientific research. Read the privacy policy of the Feline Genetics Group (only in Finnish).

For the next phrases, the answer was giving using 5-scale-Likert-scale: Strongly agree, Somewhat agree, Neither agree or disagree, Somewhat disagree, Strongly disagree, and I don't know.

1. Often seeks out physical contact from people, nudges or nuzzles *
2. Opens doors and cabinets *
3. Rests or sleeps in secure places (e.g. in laundry baskets or wardrobes) *

4. Reacts to the video or sound coming from the TV or computer screen (e.g. nature documentaries, cat videos, mouse cursor) *
5. Refuses to use the litter box if it is dirty *
6. Freezes when examined or treated by a veterinarian *
7. Seems confident at home *
8. Is comfortable and relaxed among people in social gatherings (e.g. parties) *
9. Is easily scared even by small unexpected things and sounds *
10. Defecates in inappropriate places indoors *
11. Unexpectedly scratches or bites when petted *
12. Always greets unfamiliar children visiting your home in a friendly manner (e.g. sniffs, rubs, purrs, lies on the floor) *
13. Enjoys tearing up cardboard and paper *
14. Runs or hides when hearing a sudden or loud noise coming from inside the house (e.g. vacuum cleaner, dropped object, instrument, doorbell) *
15. Runs or hides when hearing a sudden or loud noise coming from outside the house (e.g. jackhammer, fireworks) *
16. Asks to be let in and out of rooms (e.g. walks toward a door and meows) *
17. Freezes and stares intensely at nothing visible *
18. Tries to wake up members of the household early in the morning *
19. Often exhibits sudden bursts of running (“zoomies”) *
20. "Talks" to people (e.g. meows when spoken to) *
21. Shows increased activity in the early hours of morning around dawn/sunrise *
22. Chews or destroys objects when left alone at home *
23. Reacts strongly to catnip by, e.g. rubbing, rolling over or licking *
24. Chases rodents, birds, reptiles, squirrels, rabbits, insects or other small animals, given the opportunity *
25. Always purrs when petted *
26. Attempts to scratch or bite when approached by human while in possession of prey, stolen or especially delicious food *
27. Appears comfortable (confident, relaxed) when playing with familiar children *
28. Growls or hisses at a familiar cat when the familiar cat is staring, growling, or hissing at her/him *
29. Attempts to scratch or bite familiar dogs *
30. Growls or hisses when examined or treated by a veterinarian *
31. Growls or hisses at unfamiliar dogs *
32. Tends to lie on newspapers, books, keyboards or other things that are being used by people *
33. Gets excited about new toys *
34. Runs around in the house while playing *
35. Greets unfamiliar adult cats in a friendly manner (sniffs, touches noses) *
36. Growls or hisses at familiar dogs *
37. Shows restlessness or fearfulness when his/her home is modified (e.g. furniture moved from usual places, fabrics or sheets changed) *
38. Chews or eats house plants or cut flowers when given the opportunity (this does not include cat grass given for the cat to eat) *
39. Makes eye contact with familiar people, simultaneously squinting or slowly blinking its eyes *
40. Scratches claws on inappropriate objects or surfaces (e.g. couch, curtains, wallpaper) indoors *
41. Attempts to scratch or bite when petted at the base of the tail *
42. Walks to the hallway/next to front door when a household member prepares to leave the home *
43. Does not want to use the same litter box than the other cats in the household. Choose "I don't know" if he/she is the only cat in the household *

44. If the target of the cat’s aggression is inaccessible, the cat attempts to scratch or bite a “wrong” accessible target (e.g. the cat sees an unfamiliar cat through the window and bites a family member) *
45. Enjoys jumping and climbing to high places *
46. Shows restlessness (e.g. vocalizes) when a member of the household shows affection to a person *
47. Gets excited (e.g. makes chirping or chattering noises, or lashes its tail) when looking at birds or other small animals at the window *
48. Attempts to scratch or bite (in a non-playful way) when petted on the belly *
49. Carries small objects or toys in his/her mouth *
50. Attends and listens closely to everything you say or do *
51. While playing, always chases and ambushes other household members (e.g. people, dogs, or cats) playfully *
52. Growls or hisses when his/her nails are clipped *
53. Growls or hisses when given medicine by a familiar person (this does not include vets) *
54. Chases or hunts imaginary objects *
55. Attempts to scratch or bite when being bathed *
56. Growls or hisses when being bathed *
57. Shows increased activity in the evening around dusk/sunset or late at night *
58. Runs or hides from unfamiliar cats *
59. Attempts to scratch or bite when being brushed *
60. Reacts similarly in similar situations (it is possible to predict how your cat will react) *
61. Stares intensely (wide-eyed, not blinking) at unfamiliar people *
62. Exhibits self-mutilation, e.g. pulls hairs off with teeth, vigorously nibbles or bites his/her body parts *
63. Curiously investigates and explores new objects or changes in his/her environment *
64. Growls or hisses when approached by a familiar cat while eating *
65. Attempts to scratch or bite unfamiliar dogs *
66. Shows restlessness or irritation when other cats in the family are having a fight *
67. Shows excessive and intensive grooming (inhibits other behaviors) throughout the day *
68. Shows restlessness or fearfulness when unfamiliar objects are introduced into the home *
69. Plays fetch with people (retrieves thrown objects or toys) *
70. Gets along well with other cats in the household. Choose “I don't know” if he/she is the only cat in the household *
71. Seeks company of other cats in the household. Choose "I don't know" if he/she is the only cat in the household *
72. When a baby is crying, cat goes to check the baby *
73. Growls or hisses when being brushed *
74. Chases his/her own tail/rear end *
75. Attempts to scratch or bite when examined or treated by a veterinarian *
76. Rests or sleeps on top of warm appliances (e.g.TV, printer, fireplace, radiator) *
77. Attempts to scratch or bite when his/her nails are clipped *
78. Greets unfamiliar kittens in a friendly manner (sniffs, touches noses) *
79. Moves elegantly and gracefully *
80. Attempts to scratch or bite when given medicine by a familiar person (this do not include vets) *
81. Hides or escapes when a household member prepares to leave the home *
82. Urinates (crouching position) in inappropriate places *
83. Escapes or hides from unfamiliar people *
84. Is persevering, persistent and determined. Does what he/she wants even if he/she is told not to *
85. Stalks, chases or pounces on moving objects (e.g. string, balls, soft toys) *
86. Vocalizes by crying or meowing when left alone (without people) at home *
87. Sprays (standing position with tail raised vertically and vibrating) indoors (e.g. furniture, walls) *

88. Readily adapts to changes in environment or daily routines (e.g. changes in schedules, rearrangement of objects and furniture) *
89. Squirms and tries to escape when picked up and held in arms *
90. Brings prey animals into the home. If your cat has not opportunity to do this, choose “I don't know” *
91. Enjoys playing with other cats. Choose "I don't know" if he/she does not have an opportunity to play with other cats *
92. Runs or hides from unfamiliar dogs *
93. Always comes to the hallway/next to front door or is already waiting there when a household member arrives home *
94. Exhibits sudden frantic licking or chewing his/her body *
95. Comes when called *
96. Escapes or freezes when growled at, hissed at or otherwise threatened by a familiar cat *
97. Chews cables and electric wires *
98. Reacts to different events and stimuli by vocalizing (e.g. meowing) *
99. Attempts to scratch or bite a familiar cat when the familiar cat is staring, growling, or hissing at him/her *
100. Growls or hisses when petted at the base of the tail *
101. Chews or licks plastics *
102. Growls or hisses when an unfamiliar person tries to touch or pet him/her *
103. Growls or hisses at unfamiliar children *
104. Chases or follows shadows or lights (e.g. laser pointers, flashlights) *
105. Attempts to escape the home or yard/garden, if given the opportunity *
106. Is comfortable and relaxed when being petted by unfamiliar people *
107. Always greets unfamiliar adults visiting your home in a friendly manner (e.g. sniffs, rubs, purrs, lies on the floor) *
108. Active, does not spend long time idle *
109. Prefers specific types of cat litter *
110. Shows restlessness (e.g. vocalizes) when a member of the household shows affection to another pet *
111. Seeks physical contact from other cats in the household, e.g. sleeps next to them or grooms them. Choose “I don't know" if he/she is the only cat in the household *
112. Growls or hisses when approached by a familiar cat in a favorite resting place *
113. Friendly to unfamiliar dogs *
114. Growls or hisses when approached by human while in possession of prey, stolen or especially delicious food *
115. Quickly finds new ways to get attention *
116. Quickly learns to associate separate things (e.g. opening the fridge means getting food) *
117. Concentration is not disturbed by anything (e.g. other cats) during a training session. Choose "I don't know" if you do not train your cat *
118. Sucks on or chews soft fabrics or wool *
119. Growls or hisses at unfamiliar adults *
120. Purrs when sitting on someone’s lap (has jumped up voluntarily) *
121. Chases or bites people's moving legs or feet (in a non-playful way) *
122. Does not seem to recognize (e.g. hisses or escapes) a familiar cat if it smells odd (e.g. the familiar cat has visited a vet clinic). Choose "I don't know" if he/she is the only cat in the household *
123. Kneads on soft toys, fabrics, and other objects *
124. Squirms and tries to escape when picked up to the lap of a sitting/lying person *
125. Shows substrate preference when urinating or defecating in inappropriate places (e.g. laundry, bedding, carpets, flower pots). Choose "I don't know" if he/she does not urinate or defecate in inappropriate places *
126. Initiates interactive play with people (e.g. brings toys, strings, or candy wrappers to play with) *

127. Repeats the same meaningless movements (e.g. pacing back and forth next to a wall) *
128. Appears comfortable (confident, relaxed) when playing with unfamiliar people *
129. Walks tail held upright at home *
130. Does not approach people visiting his/her home right away (he/she is suspicious; however, he/she might greet people later on) *
131. Rests or sleeps in elevated places (e.g. shelves, bookcases, tops of wardrobes or cupboards) *
132. Seems comfortable and confident in unfamiliar places outside the home (e.g. cat shows) *
133. Readily responds to commands taught to him/her (e.g. sit, down, paw). Choose "I don't know" if you have not trained him/her *
134. Picky: only eats foods he/she likes and leaves other foods untouched *
135. Asks for food when hungry (e.g. meows next to his/her bowl) *
136. Shows restlessness or pacing when a household member prepares to leave the home *
137. Is fascinated by the activities of other small pets (e.g. rodents, birds, reptiles, fish) in the home *
138. Human-oriented: Enjoys the company of people more than the company of other cats *

139. If you want, you can describe your cat's behavior in detail: ________

Background and living conditions

The cat’s early life environment and experiences affect the behavior and shape the personality. The current environment may influence the behavior as well. Please take time to answer the following questions about your cat’s background. You may have to contact your cat’s breeder to be able to answer some of the questions.

Asterisk indicates mandatory field.

Data protection *
□ Hereby, I accept that my personal information, the information of my cat and all of the data collected with this questionnaire I have provided is transferred to Feline Genetics research group at the University of Helsinki and used in scientific research. Read the privacy policy of the Feline Genetics Group (only in Finnish).

---

1. How did you obtain your cat? *
o My cat was born here
o From his/her birth home (e.g. from a breeder)
o From previous a owner (not from a shelter)
o From an Animal Welfare Association/shelter as a rescue/stray
o From elsewhere

If “From elsewhere” is chosen, the following question/questions are shown:
1.1. From where did you obtain your cat? ________

If “From an Animal Welfare Association/shelter as a rescue/stray” is chosen, the following question/questions are shown:
1.2. Why had your cat come to the Animal Welfare Association/shelter? *
o He/she was a runaway house cat
o He/she was probably born to a feral cat population (no previous human contact)
o Previous owner gave him/her away
o Background of my cat is unknown

1.3. In which kind of environment was your cat kept at the Animal Welfare Association? *
o In a foster home
o In a shelter/cage

1.4. At what age did your cat come to the Animal Welfare Association? *
o My cat was born there
o As a kitten (younger than one year)
o As an adult (1 - 10 years old)
o As a senior (older than 10 years)
o I don't know

2. How old was your cat when you obtained it? *
o Under 4 months of age
o 4-6 months

o 6 months-1 year of age
o 1-1,5 years of age
o 1,5-2 years of age
o 2-3 years of age
o 3-5 years of age
o 5-7 years of age
o 7-10 years of age
o Over 10 years of age
o I don't know

3. Is your cat weaned from his/her mother or foster mother? Weaning means that the cat is permanently separated from its mother/foster mother. *
o Yes
o No, my cat is still living with his/her mother

If “Yes” is chosen, the following question/questions are shown:
3.1.When was your cat weaned? dd.mm.yyyy

3.2. If you do not remember the exact day your cat was weaned, choose the weaning age in weeks.
o I don't know
o At 1 week old
o At 2 weeks old
o At 3 weeks old
o At 4 weeks old
o At 5 weeks old
o At 6 weeks old
o At 7 weeks old
o At 8 weeks old
o At 9 weeks old
o At 10 weeks old
o At 11 weeks old
o At 12 weeks old
o At 13 weeks old
o At 14 weeks old
o At 15 weeks old
o At 16 weeks old
o At 17 weeks old
o At 18 weeks old
o At 19 weeks old
o At 20 weeks old or older

4. Did your cat have a foster mother? *
o Yes
o No

If “Yes” is chosen, the following question/questions are shown:
4.1. At which age your cat got a foster mother?
o At younger than 1 week old
o At 2 weeks old
o At 3 weeks old
o At 4 weeks old
o At 5 weeks old

o At 6 weeks old
o At 7 weeks old
o At 8 weeks old
o At 9 weeks old
o At 10 weeks old a
o At 11 weeks old a
o At 12 weeks old
o At older than 12 weeks old

5. What kind of knowledge you have about your cat's background before weaning? Please note that you can select a section to view the questions. If you cannot answer the questions, you can click the name of the section again to close it. Please try to answer as many questions as possible. *
□ Basics
□ Socialization
□ Maternal care
□ Traveling and outdoor access
□ I do not know anything about my cat's background before weaning

If "Basics" is chosen, the following question/questions are shown:
5.1. How would you describe the living conditions of your cat before weaning?
o My cat lived among people
o My cat was living outdoors or in an outdoor building, e.g. in a barn
o My cat accommodated a room or other space inside the home

5.2. What kind of food did your cat eat as a kitten (0 - 12 weeks old)? You can choose multiple options.
□ Dry food
□ Wet food
□ Raw meat/organs/bones
□ Cooked meat/organs

5.3. Did your cat eat different brands of food?
o No, only one brand of food
o Yes, from two or three different brands
o Yes, from over three different brands

5.4. How many siblings did your cat have? ________

5.5. Choose all the pet animals that lived in the same household with your cat in his/her birth home.
□ other kittens (others than the siblings of your cat)
□ other adult cats besides the mother of your cat
□ dogs
□ some other pets

If "other kittens" is chosen, the following question/questions are shown:
5.5.1. How many other kittens? ________

If "other adult cats" is chosen, the following question/questions are shown:
5.5.2. How many other adult cats? ________

If “dogs” is chosen, the following question/questions are shown:
5.5.3. How many dogs? ________

If “some other pets” is chosen, the following question/questions are shown:
5.5.4. What other pets? You can choose multiple options.
□ rodents
□ something else
□ exotic animals kept in terrarium or aquarium (e.g. reptiles, fish, insects)
□ birds
□ rabbits

If “Socialization” is chosen, the following question/questions are shown:

5.6. How often were the kittens handled between 0 - 12 weeks of age?
o Several times a day
o Daily
o Weekly
o Less than weekly

5.7. At 0 - 12 weeks of age, how often did your cat met
a) unfamiliar adults?
o Not at all
o Couple of times
o Weekly
o Daily
b) unfamiliar children?
o Not at all
o Couple of times
o Weekly
o Daily
c) unfamiliar cats?
o Not at all
o Couple of times
o Weekly
o Daily
d) unfamiliar dogs?
o Not at all
o Couple of times
o Weekly
o Daily
e) other unfamiliar animals?
o Not at all
o Couple of times
o Weekly
o Daily

If “Maternal care” is chosen, the following question/questions are shown:

5.8. Did the mother cat/foster mother nurse the kittens?
o Yes, the whole time
o Partially, but milk replacer was also needed

o Tried, but she didn’t produce milk
o Not at all

5.9. Did the mother cat/foster mother groom the kittens?
o Yes, the kittens seemed to be quite clean
o Not so well, the kittens often looked dirty or smelled bad
o Not at all

5.10. Did the mother cat/foster mother communicate with the kittens by, e.g., meowing or chirping/trilling?
o Often
o Sometimes
o No

5.11. Did the mother cat/foster mother teach the kittens (e.g. to use a litterbox)?
o Often
o Sometimes
o No

5.12. Did the mother cat/foster mother play with the kittens?
o Often
o Sometimes
o No

If “Traveling and outdoor access” is chosen, the following question/questions are shown:

5.13. At 0 - 12 weeks of age, how often did your cat travel
a) in a car?
o Not at all
o Couple of times
o Weekly
b) by public transportation (bus, train, tram, subway)?
o Not at all
o Couple of times
o Weekly

5.14. Was your cat allowed to go outdoors at the age of 0 - 12 weeks? You can choose multiple options.
□ No
□ Yes, unleashed and supervised
□ Yes, unleashed and unsupervised
□ Yes, on a leash
□ Yes, on a balcony
□ Yes, in an enclosure/cage/catio

6. If you want, you can describe your cat's background in detail. ________

7. Does your cat have offspring? *
o Yes
o No
o I don't know

If “Yes” is chosen, the following question/questions are shown:

7.1. Does your cat have a litter at this moment? *
o Yes
o No

8. Has your cat been sterilized/neutered? *
o Yes
o No
o I don't know

If “Yes” is chosen, the following question/questions are shown:

8.1. When was your cat sterilized/neutered? dd.mm.yyyy

8.2. If you do not remember the day your cat was sterilized/neutered, choose the sterilization age from the options below.
o I don't know
o Younger than 12 weeks
o 12 - 16 weeks
o 4 - 6 months
o 6 - 9 months
o 9 - 12 months
o 1 - 5 years
o Older than 5 years

8.3. What were the primary reasons to sterilize/neuter your cat? You can choose multiple answers. *
□ Inhibiting reproduction
□ Aggressive behavior
□ Inappropriate elimination
□ Some other reason
□ I don’t know, the cat was already sterilized/neutered when I got it

9. Is a hormone implant used or contraceptive pills given to your cat? *
o Yes
o No

10. What is the main reason to have this cat? *
o Family member
o Pet
o To hunt mice or other rodents in e.g. barns
o Breeding/shows
o Other reason

11. How many cats have you owned before this one? *
o this is my first cat
o 1
o 2
o 3
o 4

o 5
o 6
o 7
o 8
o 9
o 10
o 11 or more

12. Did you spend your childhood (at the age of 0 - 18 years) or part of it with cats? *
o Yes, all of it
o Yes, part of it
o No

13. How many adults are living in the same house with your cat? * ________

14. Choose all the family members and pets living in the same household with your cat. *
□ children
□ other cats
□ dogs
□ other pets
□ nothing above

If "children" is chosen, the following question/questions are shown:
14.1. How many children? * ________

If "other cats" is chosen, the following question/questions are shown:
14.2. How many other cats? * ________

If "dogs" is chosen, the following question/questions are shown:
14.3. How many dogs? * ________

If "other pets" is chosen, the following question/questions are shown:
14.4. What other pets? *
□ Rodents
□ Rabbits
□ Birds
□ Exotic animals kept in terrarium or aquarium (e.g. reptiles, fish, insects)
□ Something else

15. How many litter boxes are available for your cat (number of litter boxes your cat can access daily)? * ________

16. How big is the daily living space of your cat **in square meters**? ________

17. What kind of climbing and scratching places your cat is allowed to use? You can choose multiple answers. *
□ Large cat trees (over 1,5 meters tall)
□ Small cat trees (under 1,5 meters tall)
□ Other vertical scratching places (e.g. scratching posts, chairs your cat is allowed to scratch)
□ Horizontal scratching places (e.g. rugs, tree logs)
□ Other climbing places (e.g. closets, book shelves)
□ Nothing above

If “Large cat trees” is chosen, the following question/questions are shown:
17.1. How many large cat trees? * ________

If “small cat trees” is chosen, the following question/questions are shown:
17.2. How many small cat trees? * ________

If “vertical scratching places” is chosen, the following question/questions are shown:
17.3. How many other vertical scratching places? * ________

If “horizontal scratching places” is chosen, the following question/questions are shown:
17.4. How many horizontal scratching places? * ________

If “Other climbing places” is chosen, the following question/questions are shown:
17.5. How many other climbing places? * ________

18. On average, on how many days during one week your cat is left alone (with no people) for longer than one hour? *
o 0
o 1
o 2
o 3
o 4
o 5
o 6
o 7

19. How many hours does your cat spends alone (without people) during one week on average? * ________

20. Where does your cat normally sleeps at night? *
o On the bed with a family member
o In the bedroom, but not on the bed
o In some other room
o Confined to a specific room/area inside
o Outdoors
o I don’t know

21. What transport do you use when traveling with your cat? You can choose multiple answers. *
□ A car
□ A bus
□ A train
□ Other public transport
□ I don’t travel with my cat using any of these options

If “A car” is chosen, the following question/questions are shown:
21.1. How often do you travel with your cat by car? *
o Couple of times in a year
o Monthly
o Weekly

21.2. Does your cat have motion sickness during travels by car?
o Always

o Sometimes
o Never

If “A bus” is chosen, the following question/questions are shown:
21.3. How often do you travel with your cat by bus? *
o Couple of times in a year
o Monthly
o Weekly

21.4. Does your cat have motion sickness during travels by bus?
o Always
o Sometimes
o Never

If “A train” is chosen, the following question/questions are shown:
21.5. How often do you travel with your cat by train? *
o Couple of times in a year
o Monthly
o Weekly

21.6. Does your cat have motion sickness during travels by train?
o Always
o Sometimes
o Never

If “other public transport” is chosen, the following question/questions are shown:
21.7. How often do you travel with your cat using other public transport? *
o Couple of times in a year
o Monthly
o Weekly

21.8. Does your cat have motion sickness during travels using another public transport?
o Always
o Sometimes
o Never

22. Do you go to cat shows with your cat? *
o No, never
o Yes, less than once a year
oYes, about once a year
o Yes, couple of times in a year
o Yes, monthly or nearly monthly
o Yes, more often than monthly

23. Does your cat have free access to food at any time or does he/she get fed at specific times? *
o My cat has access to food all the time
o My cat has regular or irregular feeding times

If “My cat has regular or irregular feeding times” is chosen, the following question/questions are shown:
23.1. How many times in a day your cat is usually fed? *

o 1
o 2
o 3
o 4
o 5 or more

24. What kind of food does your cat eat? You can choose multiple options. *
□ Raw meat (including organs)
□ Raw bones and cartilage
□ Cooked meat (e.g. steamed fish, cooked chicken or minced meat)
□ Wet food
□ Dry food
□ Prey animals (e.g. mice, birds) the cat has caught
□ Something else (e.g. table scraps, home-made food)

If “Raw meat” is chosen, the following question/questions are shown:
24.1. How often does your cat eat raw meat? *
o As a main food
o Daily
o Weekly
o Less often

If “Raw bones and cartilage” is chosen, the following question/questions are shown:
24.2. How often does your cat eat raw bones and cartilage? *
o As a main food
o Daily
o Weekly
o Less often

If “Cooked meat” is chosen, the following question/questions are shown:
24.3. How often does your cat eat cooked meat? *
o As a main food
o Daily
o Weekly
o Less often

If “Wet food” is chosen, the following question/questions are shown:
24.4. How often does your cat eat wet food? *
o As a main food
o Daily
o Weekly
o Less often

If “Dry food” is chosen, the following question/questions are shown:
24.5. How often does your cat eat dry food? *
o As a main food
o Daily
o Weekly
o Less often

24.6. What brands of dry food does your cat eat? *

□ Acana
□ Almo Nature
□ Applaws
□ Arion
□ Bilanx
□ Brit Care/Brit Premium
□ Canagan
□ Carnilove
□ Concept For Life
□ Encore
□Feline Porta 21
□ Friskies
□ Hill's Prescription Diet
□ Kitekat
□ Latz
□ N&D
□ Nutrima
□ Orijen
□ Oscar
□ Perfect Fit
□ Porta 21
□ Power of Nature
□ PrimaCat
□ Purenatural
□ Purina ONE
□ Purizon
□ Royal Canin
□ Royal Canin Veterinary Diet
□ Sanabelle
□ Thrive
□ Whiskas
□ other

If “other” is chosen, the following question/questions are shown:
24.6.1. What brand of dry food does your cat eat? ________

If “Prey animals” is chosen, the following question/questions are shown:
24.7. How often does your cat eat prey animals? *
o As a main food
o Daily
o Weekly
o Less often

If “Something else” is chosen, the following question/questions are shown:
24.8. How often does your cat eat something else? *
o As a main food
o Daily
o Weekly
o Less often

25. How do you feed your cat? You can choose multiple options. *
□ From a bowl

□ With puzzle/interactive toys
□ By hiding food for the cat to find
□ With an automatic feeder
□ With something else
□ I do not feed my cat, it catches its own food

If "Something else" is chosen, the following question/questions are shown:
25.1. What do you use or how do you feed your cat? ________

26. Do you give nutritional supplements to your cat (e.g. fish oil, vitamins)? *
o Regularly
o Sometimes
o Not at all

27. Does your cat have access to outdoors? Access to outdoors includes going out in a leash, roaming freely, or being in an outdoor enclosure or a balcony. Select all matching options. *
□ Freely unsupervised
□ Freely supervised
□ In a leash
□ In a fenced backyard or outdoor cage
□ In a balcony
□ My cat does not have access to outdoors

If "Freely unsupervised" is chosen, the following question/questions are shown:
27.1. Does your cat have free access to indoors any time he/she wants? *
o Yes
o No

27.2. Does your cat go outdoors around the year or only during certain seasons? You can choose multiple answers. *
□ Around the year
□ Spring
□ Summer
□ Fall
□ Winter

27.3. On average, how many **hours** does your cat spend outdoors during one day? * ________

27.4. On average, on how many **days** a week your cat goes outdoors? *
o 0
o 1
o 2
o 3
o 4
o 5
o 6
o 7

27.5. Does your cat come indoors for the night? *
o Always
o Often
o Sometimes

o Rarely
o Never

If “Freely supervised” is chosen, the following question/questions are shown:
27.6. Do you go out with the cat around the year or only during certain seasons? You can choose multiple answers. *
□ Around the year
□ Spring
□ Summer
□ Fall
□ Winter

27.7. How often do you go out with your cat during your active outdoor season? *
o Daily
o Several times a week
o Once a week
o Less often than once a week

27.8. On average, how long do you spend outside during one visit in **minutes**? * ________

If “In a leash” is chosen, the following question/questions are shown:
27.9. Do you go out with your cat around the year or only during certain seasons? You can choose multiple answers. *
□ Around the year
□ Spring
□ Summer
□ Fall
□ Winter

27.10. How often do you go out with your cat during your active outdoor season? *
o Daily
o Several times a week
o Once a week
o Less often than once a week

27.11. On average, how long do you spend outside during one visit in **minutes**? * ________

If “In a fenced backyard or outdoor cage” is chosen, the following question/questions are shown:
27.12. Does your cat have free access to the enclosure/fenced backyard? *
o Yes, around the year
o Yes, but only when it’s warm enough
o No

27.13. Does your cat go outdoors around the year or only during certain seasons? You can choose multiple answers. *
□ Around the year
□ Spring
□ Summer
□ Fall
□ Winter

27.14. How often does your cat go to the enclosure/fenced backyard? *
o Several times a day
o Daily
o Weekly
o Less often than once a week

27.15. On average, how many **hours** does your cat spend outdoors during one day? *
____

If "In a balcony" is chosen, the following question/questions are shown:
27.16. Does your cat have free access to the balcony? *
o Yes, around the year
o Yes, but only when it's warm enough
o No

27.17. Does your cat go to the balcony around the year or only during certain seasons? You can choose more than one answer. *
□ Around the year
□ Spring
□ Summer
□ Fall
□ Winter

27.18. How often does your cat go to the balcony? *
o Several times a day
o Daily
o Weekly
o Less often than once a week

27.19. On average, how long does your cat spend on a balcony during one day in **hours**? *
____

28. How often do you play with your cat? Playing with a cat can include e.g. playing with cat toys or chasing the cat playfully/allowing the cat to chase you. *
o Several times a day
o Daily
o Several times a week
o Weekly
o Less often than weekly

29. How long do you play with your cat during one play session **in minutes**? * ________

30. What kind of toys and objects does your cat have? You can choose multiple answers. *
□ Small stuffed toys, e.g. furry mice
□ Balls with bells/crinkle balls
□ Balls without bells
□ Fishing pole toys
□ String toys
□ Feather toys (teasers)
□ Puzzles/interactive toys

□ Cardboard boxes
□ Catnip toys
□ Kick toys
□ Laser toys
□ Cat tunnels
□ Something else

If “Something else” is chosen, the following question/questions are shown:
30.1. What other toys does your cat have? ________

31. Does your cat have hobbies? You can choose multiple answers. *
□ No
□ Yes, we do agility
□ Yes, I teach tricks to my cat
□ Yes, we do scent detection/Nose Work
□ Other activity or hobby

If “Yes, we do agility” is chosen, the following question/questions are shown:
31.1. How often do you do agility? *
o Daily
o Weekly
o Monthly
o Sometimes

If “Yes, I teach tricks to my cat” is chosen, the following question/questions are shown:
31.2. How often do you teach tricks to your cat? *
o Daily
o Weekly
o Monthly
o Sometimes

If “Yes, we do scent detection/Nose Work” is chosen, the following question/questions are shown:
31.3. How often you do scent detection/Nose Work? *
o Daily
o Weekly
o Monthly
o Sometimes

If “Other activity or hobby” is chosen, the following question/questions are shown:
31.4. What other activity or hobby does your cat have? ________

31.5. How often you do this other activity/hobby?
o Daily
o Weekly
o Monthly
o Sometimes

32. Describe the temperament and behavior of your cat in a couple of sentences. ________

33. Do you feel that your cat has problematic/unwanted behaviors? *
o No
o A little

o Some
o A lot

34. Describe your cat's possible problematic/unwanted behavior. ________

35. Do you feel that your cat’s behavior has significantly changed during the past half a year? *
o Yes
o No

If “yes” is chosen, the following question/questions are shown:
35.1. How has the behavior of your cat changed? ________

Health status

Health problems, including multiple diseases and injuries can affect the behavior of your cat. Sometimes the changes in the behavior are temporary, but some health issues, for example blindness or joint pains, can change the behavior permanently. Please answer the questions in this section carefully, so that we can take the possible health problems into account when studying cat behavior.

Asterisk indicates mandatory field. You can fill this survey again anytime and it will show in available surveys.

Data protection *
□ Hereby, I accept that my personal information, the information of my cat and all of the data collected with this questionnaire I have provided is transferred to Feline Genetics research group at the University of Helsinki and used in scientific research. Read the privacy policy of the Feline Genetics Group (only in Finnish).

---

1. Is your cat deceased? *
o Yes
o No

2. How much does your cat weigh (in kilos)? ________

3. How would you describe the body condition of your cat? *
o Severely underweight
o Somewhat underweight
o Ideal weight
o Somewhat overweight
o Severely overweight
o I don’t know

4. Do you brush your cat’s teeth? *
o Yes, daily
o Yes, couple times in a week
o Yes, once a week
o Yes, less often than once a week
o Never

5. Does your cat have a congenital defect (birth defect)? *
o Yes
o No

If “yes” is chosen, the following question/questions are shown:
5.1. What kind of congenital defect does your cat have? * ________

6. Is your cat on medication at the moment? *
o Yes
o No

If “yes” is chosen, the following question/questions are shown:

6.1. What medicine is given to your cat? * ________

6.2. For what health problem is it used? * ________

7. Do you use natural products, e.g Feliway or Zylkene? *
o Yes
o No

If "yes" is chosen, the following question/questions are shown:
7.1. What natural products do you use? * ________

7.2. For what purpose are these products used? * ________

8. When was the last time your cat was taken to a veterinarian? *
o Less than 6 months ago
o 6 - 12 months ago
o 1 - 2 years ago
o 2 - 5 years ago
o Over 5 years ago
o Never

9. Are your cat's vaccinations up to date? *
o Yes
o No, my cat cat has been vaccinated but the vaccinations are overdue
o No, my cat has been vaccinated only when it was a kitten
o No, my cat has never been vaccinated
o I don't know

10. When was your cat dewormed or his/her fecal sample brought to the veterinary clinic the last time? *
o Less than 6 months ago
o 6 - 12 months ago
o 1 - 2 years ago
o 2 - 5 years ago
o Over 5 years ago
o Never

11. Has your cat had any of the following diseases or limitations? These diseases and limitations are categorized to help you find your cat's diseases/limitations. Choose the diseases or symptoms that your cat has or has had (these were shown when a categorize was selected).

SKIN, GLANDS AND FUR
□ Bald (hairless) patches

11.1. Is any disease or symptom linked to bald patches? You can choose multiple answers.
□ No
□ Allergy
□ Constipation
□ Joint pain or other pain
□ Dermatophytosis (ringworm)
□ Stress
□ Urolithiasis or urinary tract inflammation
□ Other reason

If “Other reason” is chosen, the following question/questions are shown:
11.1.1. What other reason was found for bald patches? ________

11.2. Where and how many bald patches does your cat have? ________

11.3. When was the last time your cat had these bald patches?
o At the moment
o Less than half a year ago
o More than half a year ago

□ Seborrhea (acne, stud tail, overactive sebaceous glands)
11.4. When was the last time your cat had seborrhea symptoms?
o At the moment
o Less than half a year ago
o More than half a year ago

□ Anal gland problem
11.5. When was the last time your cat had anal gland problems?
o At the moment
o Less than half a year ago
o More than half a year ago

□ Atopy
11.6. When was the last time your cat had atopy symptoms?
o At the moment
o Less than half a year ago
o More than half a year ago

□ Other problems of the skin, glands or fur (e.g. severe dandruff)
11.7. What other skin, gland or fur problem does your cat have? ________

11.8. When was the last time your cat had this problem/disease?
o At the moment
o Less than half a year ago
o More than half a year ago

EYES

□ Blind
11.9. When was your cat’s blindness noticed? Choose “I don’t know” if your cat was already blind when you acquired him/her.
o My cat has been blind from birth
o Less than 3 months ago
o 3 - 6 months ago
o 6 - 12 months ago
o More than a year ago
o I don’t know

11.10. How was blindness diagnosed?
o By a veterinarian
o By myself, based on the behavior of the cat

□ Eye/eyes removed/missing
11.11. Were both eyes removed or just one?
o Just one
o Both

11.12. When were the eye/eyes removed? Choose “I don’t know” if the eye/eyes were already removed when you acquired him/her.
o My cat has been missing an eye/eyes from the birth
o Less than 3 months ago
o 3 - 6 months ago
o 6 - 12 months ago
o More than a year ago
o I don’t know

□ Eye infection

11.13. When was the last time your cat had an eye infection?
o At the moment
o Less than half a year ago
o More than half a year ago

□ Blocked tear duct

11.14. When was the last time your cat had symptoms caused by a blocked tear duct?
o At the moment
o Less than half a year ago
o More than half a year ago

□ Missing tear duct
□ Other eye-related disease/problem

11.15. What other eye-related disease/problem does your cat have? ________

11.16. When was the last time your cat had this other eye disease/problem?
o At the moment
o Less than half a year ago
o More than half a year ago

EARS

□ Deaf

11.17. Is your cat deaf from both ears?
o Yes
o No, just from one

11.18. When did your cat become deaf? Choose “I don’t know” if your cat was already deaf when you acquired him/her.
o My cat has been deaf from birth
o Less than 3 months ago
o 3 - 6 months ago
o 6 - 12 months ago
o More than a year ago
o I don’t know

11.19. How was deafness diagnosed?
o By a veterinarian, using BAER
o By a veterinarian, using another method
o By myself, based on the behavior of my cat

□ Ear infection

11.20. When was the last time your cat had an ear infection?
o At the moment

o Less than half a year ago
o More than half a year ago

□ Other ear-related disease/problem

11.21. What other ear-related disease/problem does your cat have? ________

11.22. When was the last time your cat had this other ear disease/problem?
o At the moment
o Less than half a year ago
o More than half a year ago

MOUTH AND TEETH

□ Dental calculus (tartar)

11.23. When was the last time your cat had dental calculus?
o At the moment
o Less than half a year ago
o More than half a year ago

11.24. How much dental calculus does your cat have?
A little
Moderately
A lot

□ Gingivitis (inflammation of gum tissue)

11.25. When was the last time your cat had gingivitis?
o At the moment
o Less than half a year ago
o More than half a year ago

□ Stomatitis (inflammation of the mouth)

11.26. When was the last time your cat had stomatitis?
o At the moment
o Less than half a year ago
o More than half a year ago

□ Tooth resorption (TR; previously Feline odontoclastic resorption lesion, FORL)

11.27. When was the last time TR was noticed or your cat had TR symptoms?
o At the moment
o Less than half a year ago
o More than half a year ago

□ Other mouth or teeth-related disease/problem (e.g. crossbite, missing teeth)

11.28. What other mouth or teeth-related disease/problem does your cat have? ________

11.29. When was the last time your cat had this other mouth/teeth disease/problem?
o At the moment
o Less than half a year ago
o More than half a year ago

KIDNEYS AND URINARY TRACT

□ Urinary tract infection

11.30. When was the last time your cat had a urinary tract infection?
o At the moment
o Less than half a year ago

o More than half a year ago
□ Idiopathic cystitis (FIC)

11.31. When was the last time your cat had symptoms of idiopathic cystitis (e.g. inappropriate elimination)?
o At the moment
o Less than half a year ago
o More than half a year ago

11.32. Have you noticed some situations that trigger the symptoms? Please describe.
____

□ Bladder stones (urine crystals, uroliths)
11.33. When was the last time your cat had bladder stones?
o At the moment
o Less than half a year ago
o More than half a year ago
□ Kidney failure
11.34. Is your cat's kidney failure acute or chronic?
o Acute
o Chronic

11.35. When was the kidney failure diagnosed?
o Less than half a year ago
o More than half a year ago

11.36. When was the last time your cat had symptoms of kidney failure?
o At the moment
o Less than half a year ago
o More than half a year ago
□ Other kidney or urinary tract related disease/problem
11.37. What other kidney or urinary tract related disease/problem does your cat have?
____

11.38. When was the last time your cat had this other kidney or urinary tract disease/problem?
o At the moment
o Less than half a year ago
o More than half a year ago

HEATH, BLOOD AND BLOOD VESSELS

□ HCM (hypertrophic cardiomyopathy)
11.39. When was HCM diagnosed?
o Less than half a year ago
o More than half a year ago

11.40. What symptoms of HCM does your cat have? ________
□ Heart murmur
11.41. When was the heart murmur noticed last time?
o Less than half a year ago
o More than half a year ago

□ Other heart, blood or blood vessel related disease/problem

11.42. What other heart, blood or blood vessel related disease/problem does your cat have? ________

11.43. When was the last time this other heart, blood or blood vessel disease/problem caused symptoms to your cat?
o At the moment
o Less than half a year ago
o More than half a year ago

MUSCULAR AND SKELETAL SYSTEM

□ Tail kink (crooked/bent tail)

11.44. Do you think that tail kink affects the behavior of your cat?
o Yes
o No

If "yes" is chosen, the following question/questions are shown:
11.44.1 How does tail kink affect the behavior of your cat? ________

□ Hip dysplasia or other deformation of bones/joints

11.45. Does hip dysplasia or other deformation of bones/joints cause symptoms to your cat?
o No
o Minor symptoms
o Major symptoms

□ Osteoarthritis or other joint pain

11.46. What symptoms does osteoarthritis/other joint pain cause? Please, choose all symptoms, that your cat shows at least some times.
□ Withdraw/hide
□ Playing with other cats decreased
□ Limping
□ Reluctant to jump up/down
□ Reluctant to move
□ Decreased grooming
□ More irritable
□ No symptoms

□ Amputation

11.47. What was amputated?
o One front leg
o Both front legs
o One hind leg
o Both hind legs
o Whole tail
o Tip of the tail or part of the tail
o A toe
o Several toes
o Something else

If "yes" is chosen, the following question/questions are shown:
11.47.1. What was amputated? ________

11.48. When did your cat had amputation surgery?
o Less than half a year ago

o Half a year ago - year ago
o More than a year ago
o I don’t know

□ Injury caused by a traumatic accident (e.g. traffic accident or falling from a window)

11.49. What kind of injury does your cat have? How does it affect the behavior of your cat?
____

□ Other muscular or skeletal system related disease/problem

11.50. What other muscular or skeletal system related disease/problem does your cat have?
____

11.51. When was the last time this other muscular or skeletal system related disease/problem caused symptoms to your cat?
o At the moment
o Less than half a year ago
o More than half a year ago

DIGESTIVE SYSTEM

□ Repetitive vomiting (this does not include vomiting of hairballs)

11.52. How often does your cat vomit?
o Daily
o Weekly
o Less than once a week

□ Repetitive diarrhea

11.53. How often does your cat have diarrhea?
o Daily
o Several times a week
o Once a week
o Less than once a week

□ Repetitive constipation

11.54. How often does your cat have constipation?
o Constantly
o Weekly
o Monthly
o Less than monthly

□ Acute inflammatory bowel disease

11.55. When was the last time your cat had an acute inflammatory bowel disease?
o At the moment
o Less than half a year ago
o More than half a year ago

□ Chronic inflammatory bowel disease

11.56. When was the chronic inflammatory bowel disease diagnosed?
o Less than half a year ago
o More than half a year ago

□ Other digestive system related disease/problem

11.57. What other digestive system related disease/problem does your cat have?
____

11.58. When was the last time your cat had this digestive system related disease/problem?
o At the moment
o Less than half a year ago

o More than half a year ago

RESPORATORY SYSTEM

□ Asthma

11.59. What symptoms does asthma cause to your cat? ________

11.60. How often does your cat have symptoms of asthma?
o Daily
o Weekly
o Less than weekly

□ Respiratory infection

11.61. When was the last time your cat had a respiratory infection?
o At the moment
o Less than half a year ago
o More than half a year ago

□ Other respiratory system related disease/problem

11.62. What other respiratory system related disease/problem does your cat have? ________

11.63. When was the last time your cat had this other respiratory system related disease/problem?
o At the moment
o Less than half a year ago
o More than half a year ago

NERVOUS SYSTEM

□ Epilepsy

11.64. When was the last time your cat had an epileptic seizure?
o Less than a week ago
o One week - a month ago
o A month - 6 months ago
o Over 6 months ago

11.65. How often does your cat have epileptic seizures?
o Daily
o Weekly
o Couple times in a month
o Monthly
o Less often than monthly

□ Nystagmus (involuntary eye movement, “dancing eyes”)

11.66. How does nystagmus affect your cat’s life? ________

11.67. When was the last time nystagmus was noticed?
o At the moment
o Less than half a year ago
o More than half a year ago

□ Other nervous system related disease/problem

11.68. When was the last time this other nervous system related disease/problem caused symptoms to your cat?
o At the moment

o Less than half a year ago
o More than half a year ago

11.69. What other nervous system related disease/problem does your cat have? ________

REPRODUCTIVE ORGANS

□ Uterine infection (pyometra)
11.70. When was the uterine infection noticed?
o Less than a month ago
o A month – 6 months ago
o More than 6 months ago
□ Undescended testis (cryptorchidism)
11.71. Does your cat have an undescended testis at this moment?
o Yes
o No
□ Other reproductive disease or problem
11.72. When was the last time your cat had this other reproductive related disease/problem?
o Less than a month ago
o A month – 6 months ago
o More than 6 months ago

11.73. What other reproductive disease or problem does your cat have? ________

HORMONES AND METABOLISM
□ Hyperthyroidism
11.74. When was your cat's hyperthyroidism diagnosed?
o Less than half a year ago
o More than half a year ago

11.75. When was the last time hyperthyroidism caused symptoms to your cat?
o At the moment
o Less than half a year ago
o More than half a year ago
□ Diabetes mellitus
11.76. When was your cat's diabetes diagnosed?
o Less than a month ago
o Less than six months ago
o Over six month ago

11.77. When was the last time your cat had symptoms of diabetes?
o At the moment
o Less than half a year ago
o Moren than half a year ago

11.78. If your cat currently has symptoms of diabetes, please describe them. ________
□ Other hormone or metabolism related disease/problem
11.79. When was the last time this other hormone or metabolism related disease/problem caused symptoms to your cat?
o At the moment
o Less than half a year ago

o More than half a year ago

11.80. What other hormone or metabolism related disease/problem does your cat have? ________

ALLERGIES AND AUTOIMMUNE DISEASES

□ Food allergy

11.81. What symptoms does food allergy cause to your cat? You can choose multiple options.
□ Skin symptoms
□ Ear redness/itching/infection
□ Gastrointestinal symptoms
□ Some other symptoms

If “Some other symptoms”:
11.81.1. What other symptoms does food allergy cause to your cat? ________

□ Other allergy or autoimmune disease

11.82. What allergy or autoimmune disease does your cat have? ________

11.83. When was this allergy or autoimmunity disease diagnosed?
o Less than a month ago
o A month - 6 months ago
o Over 6 months ago

11.84. What symptoms does this other allergy or autoimmunity disease cause to your cat? ________

CANCERS AND TUMORS

□ Mammary tumour

11.85. Has the mammary tumor been surgically removed from your cat?
o Yes
o No

11.86. What symptoms does mammary tumor cause to your cat? ________

□ Other cancer or tumor

11.87. What other cancer or tumor does your cat have? ________

11.88. Has this cancer or tumor been surgically removed from your cat?
o Yes
o No

11.89. What symptoms does the cancer or tumor cause to your cat? ________

PARASITES AND PROTOZOANS

□ Internal parasites/intestinal parasites (e.g. tapeworms, roundworms)

11.90. When were the internal parasites noticed the last time?
o Less than a week ago
o Less than half a year ago
o More than half a year ago

□ External parasites (e.g. fleas, ticks, ear mites)

11.91. When were the external parasites noticed the last time?
o Less than a week ago
o Less than half a year ago
o More than half a year ago

11.92. What external parasites your cat has had within the last month?
□ *Demodex* mites
□ Earmites
□ Lice
□ Fleas
□ *Cheyletiella* mites
□ *Notoedres* mites
□ Ticks

□ Protozoans (e.g. *giardia, toxoplasma gondii*)
11.93. When were the protozoans noticed the last time?
o Less than a week ago
o Less than half a year ago
o More than half a year ago

OTHER DISEASES AND HEALTH PROBLEMS

□ Feline upper respiratory infection (URI, cat flu)
11.94. When was the last time your cat had a respiratory infection?
o At the moment
o More than half a year ago
o Less than half a year ago

11.95. Has the causing pathogen been identified?
□ No
□ Yes, as feline calicivirus
□ Yes, as feline herpesvirus
□ Yes, as mycoplasma
□ Yes, as chlamydia
□ Yes, as bordetella

□ FIP (feline infectious peritonitis)
11.95. How does FIP affect your cat? What kind of symptoms does the cat have? ________

□ FIV (feline immunodeficiency virus)
11.96. How is FIV presented in your cat?
o asymptomatic
o minor symptoms
o severe symptoms

□ FeLV (feline leukemia virus)
11.97. Does your cat have symptoms caused by FeLV?
o Yes
o No

□ Feline hyperesthesia syndrome (rolling skin disease)
11.98. When was the last time your cat had a hyperesthesia episode?
o Less than half a year ago
o Half a year ago - one year ago
o More than a year ago

11.99. How often does your cat have hyperesthesia episodes?
o Daily
o Weekly
o Monthly
o Less often than monthly

12. If you did not find your cats disease or health problem, describe it below. ________
